# Improving the Biostability of Extra Virgin Olive Oil with Olive Fruit Extract During Prolonged Deep Frying

**DOI:** 10.3390/foods14020260

**Published:** 2025-01-15

**Authors:** Taha Mehany, José M. González-Sáiz, Consuelo Pizarro

**Affiliations:** Department of Chemistry, University of La Rioja, 26006 Logroño, Spain; taha.abdellatif@unirioja.es (T.M.); josemaria.gonzalez@unirioja.es (J.M.G.-S.)

**Keywords:** oxidation, deep frying stability, thermal degradation, natural antioxidants, bitterness, verbascoside, secoiridoids, sensory attributes, phenolic compounds, tyrosol, healthy food

## Abstract

This study explores approaches to enhancing the biostability of extra virgin olive oil (EVOO) supplemented with olive fruit extract (OFE) enriched with hydroxytyrosol (HTyr). The investigation focuses on prolonged deep frying (DF) conditions at 170 °C and 210 °C, over durations ranging from 3 to 48 h, with the aim of improving sensorial attributes, polyphenolic content, and thermal oxidative stability. Parameters, such as acidity, peroxide value (PV), K_232_, K_270_, ΔK, phenolic compounds, and sensory attributes, were monitored. The PV did not exceed the standard limit in HTyr-EVOO at 210 °C/24 h; however, in non-supplemented EVOOs, it remained within the limits only up to 210 °C/18 h. Acidity stayed within the acceptable limit (≤0.8) at 170 °C/24 h in both enriched and non-enriched EVOOs. K_232_ values were ≤2.5 in HTyr-EVOO fried at 170 °C/18 h. K_270_ and ΔK did not exceed the limits in HTyr-EVOO at 170 °C/3 h, whereas they surpassed them in non-supplemented oils. Additionally, HTyr and tyrosol levels were significantly higher (*p* < 0.05) in HTyr-EVOOs. Phenolic compounds, including verbascoside, pinoresinol, 1-acetoxypinoresinol, and phenolic acids, such as chlorogenic, vanillic, homovanillic, 4-dihydroxybenzoic, and caffeic acids, were detected in HTyr-EVOOs. Oxidized secoiridoid derivatives increased significantly as DF progressed. Moreover, sensory analysis revealed that positive attributes in EVOOs—such as fruity, bitter, and pungent notes—decreased significantly with increasing temperature and frying duration (*p* < 0.05). Beyond 210 °C/6 h, these attributes were rated at zero. However, HTyr-EVOOs exhibited lower rancidity compared to non-enriched oils under identical conditions, attributed to the protective effect of HTyr. In conclusion, HTyr-EVOOs demonstrated thermal stability up to 210 °C/6 h, retaining desirable sensory qualities, higher phenolic content, and reduced degradation. These findings indicate that natural OFEs have strong potential as food additive in deep fried EVOOs, enhancing sensory properties, health benefits, and overall oil stability. This innovation provides a practical solution for the food industry by improving the biostability and versatility of EVOO. Further research is recommended to investigate various EVOO categories and oils from diverse origins.

## 1. Introduction

Extra virgin olive oil (EVOO), a staple of the Mediterranean diet, is renowned for its unique sensory properties and exceptional health benefits, such as its anti-diabetic, anti-atherogenic, anti-hepatotoxic, anticancer, antioxidant, anti-hypertensive, and anti-obesity effects [1,2]. Numerous studies conducted over the past ten years have consistently demonstrated that following a Mediterranean diet rich in olive oil is associated with reduced mortality rates and increased lifespan [3,4]. The stability and nutritional properties of edible oils depend strongly on their chemical composition. In fact, EVOO is considered a stable and healthy edible oil because it contains relatively more unsaturated fatty acids (UFAs) in the saponifiable fraction (major compounds), particularly mono-unsaturated fatty acids (MUFAs) in the form of oleic acid (OA), which constitutes the primary component of EVOO at a ratio of 70–80% [5,6]. In addition, OA exerts beneficial anti-inflammatory effects, improves immune system function, and reduces cholesterol levels and blood pressure [5,6]. The unsaponifiable fraction of EVOO comprises a group of minor components that represent about 1–2% of the oil weight and contains over 200 chemical compounds, such as polyphenols, tocopherols, hydrocarbons, chlorophyll, squalene, pigments, volatile compounds, beta-carotene, esters, alcohols, and sterols, among others [7]. Moreover, minor components, e.g., phytosterols, tocopherols, squalene, and phenolic constituents, enhance both the flavor and health benefits of EVOO. As a result, the organoleptic characteristics, oxidative stability, and biological properties of EVOO are attributed to a synergy of components, with OA being the main ingredient, followed by the bioactive minor constituents [8,9].

The most vital minor components of EVOOs include polyphenols. Phenolic compounds in EVOOs have numerous health benefits, such as playing a critical role in preserving genomic stability. This is primarily due to their ability to shield both nuclear and mitochondrial DNA from damage induced by oxidative stress. Additionally, polyphenolic compounds help prevent mitochondrial dysfunction by supporting the body’s internal antioxidant systems [10]. Furthermore, phenolics have also been found to postpone cellular aging and mitigate disruptions in intercellular communication pathways. This effect may occur by modulating chronic inflammation, such as that associated with steatohepatitis, which is closely linked to the aging process [11]. Another study investigated the impact of daily EVOO intake on oxidative stress and antioxidant defenses in elderly individuals living in institutional settings. Over six weeks, 50 mL of polyphenol-rich EVOO was shown to reduced total cholesterol, low-density lipoproteins (LDL), and triglycerides while increasing high-density lipoproteins (HDL) and antioxidant capacity, as well as improving plasma biomarkers [12]. The phenolic fraction of EVOO is characterized by significant heterogeneity, with at least 36 different phenolic compounds identified in its composition. The variability in its chemical composition arises from differences in cultivars, extraction, production, and storage techniques, as well as diverse environmental conditions, which are key factors in determining its quality [9].

According to numerous investigations, the health effects of EVOOs are mostly attributable to the main secoiridoid derivatives, such as oleuropein (Ole), oleocanthal (Oc), oleacein, and simple phenols, such as HTyr and tyrosol (Tyr) [13,14]. Interestingly, HTyr exhibits several health benefits, such as antimicrobial, antithrombotic, hypolipidemic, hypoglycemic, anti-aging, anticancer, anti-inflammatory, and cardioprotective effects [15]. Moreover, HTyr enhances cellular antioxidant defenses and causes a decrease in oxidative damage [16], as well as improving skin wound healing in diabetic mice [17]. Recently, HTyr has emerged as the most significant antidiabetic nutraceutical component in EVOOs [18]. In support of this, a recent study examined the anti-diabetic effects of EVOO, particularly focusing on HTyr. Additionally, EVOO and HTyr were found to enhance liver and muscle insulin receptor expression, improve energy balance, and protect against oxidative stress. Molecular analyses further revealed HTyr binding with PGC-1α, IRE-1α, and PPAR-γ, suggesting potential for drug development [19]. Based on the large number of clinical studies, the European Food Safety Authority (EFSA) authorized a claim about the benefits of the daily ingestion of olive oil. The EFSA panel proposed the intake of 5 mg/day of HTyr and its derivatives to obtain these effects. These quantities can easily be obtained within the context of a Mediterranean diet and the consumption of moderate amounts of olive oil [15]. Additionally, HTyr is extensively metabolized by the gut microbiota, which transforms part of the absorbed HTyr into hydroxylated phenylacetic acids, 2-(4′-hydroxyphenyl) acetic acid, and 2-(3′,4′-dihydroxyphenyl) acetic acid [20].

Deep frying (DF) is among the most ancient and widely practiced culinary methods globally, renowned for its ability to create flavorful, stable, and easily prepared dishes. This technique involves submerging food in hot oil, typically at temperatures between 160 and 180 °C, facilitating both heat and mass transfer. The degree of oil absorption in foodstuffs is primarily influenced by the reduction in internal pressure caused by moisture evaporation [21,22,23]. Furthermore, the oil absorption in fried foods tends to increase with higher levels of polyunsaturated fatty acids (PUFAs), lower frying temperatures, extended cooking durations, and specific food characteristics, such as being flat, porous, or moist [21,22,23]. Moreover, during DF, oils can deteriorate due to the accelerated autoxidation of triglycerides and hydrolytic alterations, processes strongly influenced by the type of oil and its fatty acid composition (FAC) [24,25]. High temperatures during DF alter the FAC, reducing the content of PUFAs while increasing the levels of SFAs and trans fatty acids (TFAs). Oils rich in PUFAs are more prone to peroxidation and the formation of lipid oxidation products (LOPs), such as aldehydes, compared to oils rich in MUFAs, like EVOO and SFAs. LOPs can potentially penetrate food and pose health risks when consumed [26,27,28].

Recently, Harzalli et al. [29] reported that sunflower oil enriched with olive by-product extracts, such as leaves and olive mill wastewater, reduced the lipid oxidation of potatoes during frying and improved the quality, safety, and nutritional value of fried food. In addition, Vidal et al. [30] reported that phenolic compounds significantly contribute to the taste and aroma of industrially processed virgin olive oil (VOO) due to the strong correlation between polyphenols and organoleptic properties. Therefore, we hypothesize that supplementing EVOO with exogenous polyphenols could be a promising strategy for the agro-food industry, enhancing health benefits while serving as a natural antioxidant and stabilizer from a technological perspective. To the best of our knowledge, there are no existing reports regarding the optimum prolonged DF conditions for achieving thermally stable EVOO with low sensorial defects, high sensorial positive attributes, low degradation, and high phenolic content, when supplemented with olive fruit extract (OFE) enriched with HTyr and its derivatives. Therefore, this study aims to monitor the oxidation and degradation of EVOO enriched with HTyr and its derivatives when exposed to prolonged and continuous DF conditions at 170 °C and 210 °C (from 3 to 48 h). Additionally, sensorial qualities and phenolic profiles are also assessed.

## 2. Materials and Methods

### 2.1. Materials

The following chemicals and reagents were used in the present study: ethanol (≥99.93%), sodium hydroxide (0.1 mol/L or 0.1 N), chloroform (≥99%), and cyclohexane (≥99.8%) were supplied by VWR (VWR International, LLC, 1–3 Rue d’Aurion, Rosny-sous-Bois, France). Glacial acetic acid with ≥99% purity was supplied by Fisher Scientific Ltd. (Loughborough, UK). Diethyl ether (≥99.8%) was purchased from Honeywell Riedel-de Haen GmbH, Seelze, Germany. Potassium iodide (≥99.0%), phosphoric acid (H_3_PO_4_) (49–51%), and sodium thiosulfate (Na_2_S_2_O_3_.5H_2_O) with 99% purity were purchased from Sigma-Aldrich (Saint Louis, MO, USA). HPLC-grade syringic acid ≥97%, and tyrosol (2-(4-hydroxyphenyl) ethanol) with ≥98% purity were supplied by Sigma-Aldrich Chemie GmbH (Steinheim, Germany). LC-MS-grade methanol with ≥99.9% purity and acetonitrile with 100% purity were supplied by Fisher Scientific Ltd. (Loughborough, UK). Ultrapure water was obtained from a MilliQ system (Millipore, Bedford, MA, USA).

For sensory analyses, watch glasses, odorless markers to mark the tasting glasses, tasting sheets, bottled water, apples, and standard cups were used following the standard procedure of the International Olive Council [31]; additionally, an air oven was used (Super M 1072, Barcelona, Spain) to maintain the temperature of the sample at 28 ± 2 °C before the analysis. Moreover, a balance (Sartorius AG120S, Göttingen, Germany) with a precision of +0.1 g, a thermometer for controlling the room temperature, and a thermometric probe (J. P. SELECTA, Barcelona, Spain) to measure the temperature of the samples were used.

### 2.2. Olive Oil and Olive Fruit Extract Sampling

EVOO cv. Manzanilla Cacereña was sourced from Aceite Artajo, Navarra, Spain. The Manzanilla cultivar was chosen for this study over other Spanish EVOO varieties due to its distinctive sensory attributes, including bitterness, pungency, and fruitiness, as well as its moderate polyphenol content, as demonstrated by preliminary analyses. Additionally, dried OFE with 20% hydroxytyrosol (HTyr) was obtained from Natac BioTech (Natac, Madrid, Spain). The extract was stored at 7 ± 2 °C for further analyses.

### 2.3. Supplementation of EVOO with HTyr

HTyr was used to reinforce olive oil samples derived from olive fruit extract (OFE) as an exogenous natural polyphenol source, up to 650 mg/kg. This concentration was chosen to mimic oils that naturally have a high polyphenol content. OFE is a natural source of hydroxytyrosol and its derivatives. Due to the polarity of hydroxytyrosol, exogenous OFEs are water soluble and only slightly soluble in oil. As a result, OFE was first subjected to solubilization in water, and then this aqueous emulsion was used to reinforce the oils. After centrifugation, two phases were obtained, namely the supernatant, containing the supplemented EVOO, and the precipitate, comprising the aqueous phase. In details, the preparation of supplemented EVOO with HTyr up to 650 mg/kg was carried out as follows: first, 40 g of OFE extract was added to 400 g of H_2_O. Then, the solution was stirred using a magnetic stirrer (IKA-WERKE, Staufen, Germany) at room temperature (RT) for 30 min. Subsequently, 200 g of this aqueous solution was mixed with 500 g of Manzanilla oil, and it was stirred mechanically at RT for 60 min. The prepared solution was then centrifuged (Sorvall RC-6 Plus, Osterode, Germany) at 9961× *g* for 20 min. Finally, the supernatant containing supplemented EVOO cv. Manzanilla was transferred into an amber container and preserved at 7 ± 2 °C for further analyses [32]. In this regard, Figure S1 illustrates the detailed supplementation process of EVOO cv. Manzanilla with olive fruit extract rich in hydroxytyrosol and its derivatives, resulting in the production of EVOO for further use and analyses.

### 2.4. Deep Frying Experiment

Both non-supplemented and supplemented EVOO samples with HTyr were subjected to deep frying at two different temperatures, i.e., 170 and 210 °C, for various times, i.e., 3, 6, 12, 18, 24, and 48 h, to obtain 24 different experiments (Table 1). An oil sample from each experiment was heated in a 0.5 L volume flask with a Soxhlet system (J. P. SELECTA, Barcelona, Spain) and equipped with an Electtemp-basic (J. P. SELECTA, Barcelona, Spain) to control the time and temperature. For the DF process, 0.4 L of oil was placed in the fryer and subjected to continuous heating at 170 ± 10 °C for 3, 6, 12, 18, 24, and 48 h as well as at 210 ± 10 °C for 3, 6, 12, 18, 24, and 48 h. During the frying experiments, the DF process was not repeated, nor was additional oil added. After the DF process, the samples were allowed to cool, and they were then stored at 5 °C in an amber glass for further analyses.

### 2.5. Quality Parameters of EVOOs

The free acidity (% oleic acid), peroxide value (PV) expressed as (mEq O_2_/kg), and UV absorption coefficients at 270 nm (K_270_), 232 nm (K_232_), and ΔK were determined according to the methodology described in the European Commission Regulation EEC 2568/91 [33].

#### 2.5.1. Acidity

A total of 5 g of EVOO sample was dissolved with 25 mL of ethanol/diethyl ether (1:1, *v*/*v*), and 1% phenolphthalein was used as an indicator. Then, the solution was titrated with NaOH solution (0.1 N) under shaking until the appearance of a faint pink solution was detected. The free acidity was calculated as % oleic acid according to the following equation:

(1) $$Acidity(\%\;of\;oleic\;acid)=\frac{V\times N\times 28.2}{m}\;$$

where V is the volume of NaOH consumed (mL), N is the normality of NaOH, and m is the mass of EVOO (g).

#### 2.5.2. Peroxide Value

A total of 5 g of EVOO sample was prepared into a flask with 30 mL of chloroform/acetic acid solution (3:2, *v*/*v*); then, 0.5 mL of saturated aqueous potassium iodide solution and 30 mL of distilled water were added while shaking the solution vigorously. Then, the mixture was titrated with 0.1 N of sodium thiosulfate (Na_2_S_2_O_3_.5H_2_O) solution in the presence of starch solution indicator (prepared from 1 g starch/100 mL H_2_O) until the disappearance of the blue color or the detection of a white milky color. PV was calculated using the following equation:

(2) $$PV(mEq\;O_{2}/kg)=\frac{V\times N\times 1000}{m}\;$$

where V is the volume (mL) of Na_2_S_2_O_3_.5H_2_O (blank corrected), N is the normality of sodium thiosulfate (0.1 N), and m is the mass of EVOO (g).

#### 2.5.3. Spectrophotometric Characteristics

The conjugated trienes’ (K_270_) and conjugated dienes’ (K_232_) absorption coefficients were measured using a 1% EVOO in cyclohexane solution (0.1 g of oil sample–10 mL of cyclohexane, *w*/*v*) in a quartz cuvette with a 1 cm (10 mm) path length. When measuring, a blank sample was firstly made with the used solvent only (cyclohexane). The values of these absorptions were determined using a UV–Vis spectrophotometer (model 8453 Hewlett Packard, Waldbronn, Germany) and expressed as specific extinction coefficients, calculated by the following equation:

(3) $$K\lambda =\frac{Ab\lambda }{C\times L}$$

where Kλ is the spectrophotometric absorption in the ultraviolet region (232 or 270), Ab is the absorbance, C is the concentration of the prepared solution in g/100 mL (1.0%), and L is the cuvette path length (10 mm).

The spectrophotometric test of olive oil requires the determination of ΔK, as defined using the following equation:

(4) $$\Delta K=\frac{K270-((K266)+(K274))}{2}$$

### 2.6. Extraction of Phenolic Compounds

Approximately 2.0 g of each deep fried olive oil experimental sample and non-fried EVOOs (controls) were placed in 10 mL screw-cap test tubes. Then, 1 mL of the internal standard solution (syringic acid) was added to the previously weighed sample. The tubes were sealed with the screw cap and shaken vigorously for exactly 30 s at RT using a shaker (Heidolph, D-91126, Schwabach, Germany). Next, 5 mL of the methanol/water (80/20, *v*/*v)* extraction solution was added to each tube. Then, the samples were shaken robustly again for 1 min. Additionally, the samples were sonicated in an ultrasonic bath for 15 min at 30 °C (Ultrasons, J. P. SELECTA, Barcelona, Spain) equipped with a shaker (Heidolph, RZR1, Schwabach, Germany), and water bath to adjust the extraction temperature (Julabo, F25, Seelbach, Germany). Finally, the ultrasonicated and extracted samples were centrifuged (Gerätebau Eppendorf 5403 Refrigerated Centrifuge, Eppendorf GmbH, Hamburg, Germany) at 4193× *g*/25 min. Then, an aliquot from the supernatant phase was filtered through a 5 mL plastic syringe, with a 0.45 µm PVDF filter (Millex HV, Merck Millipore Ltd., Cork, Ireland), for further injection for HPLC analysis [34].

### 2.7. High-Performance Liquid Chromatography (HPLC) Analysis

#### 2.7.1. Preparation of External Calibration Standards and Validation of the Method

Subsequently, 0.030 g of tyrosol (external standard) and 0.015 g of syringic acid (internal standard) were accurately weighed into a 10 mL volumetric flask, and the volume was adjusted with a methanol/water solution (80:20, *v*/*v*). A 100 µL aliquot of this solution was then transferred to another 10 mL volumetric flask, and the volume was similarly adjusted with the methanol/water solution (80:20, *v*/*v*). The resulting external calibration solution had concentrations of 0.030 mg/mL for tyrosol and 0.015 mg/mL for syringic acid. This solution remains stable for up to three months when stored at +4 °C in a refrigerator.

A standard tyrosol stock solution (1 mg/mL) was prepared by dissolving 10 mg of tyrosol in 10 mL of methanol/water solution (80:20, *v*/*v*). This stock solution was used to create a series of standards ranging from 0.030 to 0.090 mg/mL.

The calibration curve for the HPLC analysis was constructed by plotting the ratio of the tyrosol peak area against its concentration levels (Figure S2). The peak area was proportional to the tyrosol concentration, and the calibration curve was described by the following regression equation: y = 10915x + 611.2 (R^2^ = 0.9986), where x represents the concentration of tyrosol (mg/mL) and y represents the peak area. The peak area showed a linear relationship with tyrosol concentration within the range of 0.030–0.090 mg/mL.

The calculated limits of detection (LOD) and quantification (LOQ) were 0.0098 mg/kg and 0.0298 mg/kg, respectively.

Regarding accuracy (trueness and precision), each oil sample was measured in triplicate. The standard deviation was calculated to assess the closeness of agreement among a series of measurements obtained from multiple samplings of a homogeneous sample. This evaluation ensured the method’s precision, reproducibility, and repeatability, as well as the reliability of the results.

#### 2.7.2. HPLC Condition and Analysis

Chromatographic analysis was performed using a Hewlett Packard 1100 series system (Agilent Technologies, Hewlett-Packard-Straße 8, Waldbronn, Germany) equipped with a high-pressure gradient pump, a photodiode array detector (DAD), an autosampler, and a degasser. The chromatographic separation was carried out using a Spherisorb octadecyl silyl 2 (ODS) chromatographic column (250 mm × 4.6 id, 5.0 μm particle size, Waters, Dublin, Ireland), maintained at 25 °C and protected by a guard column of the same material. A total of 20 µL of each sample was injected and analyzed in triplicate.

In detail, the UV spectrophotometer was switched on 1 h before analysis. The mobile phase consisted of 0.2% H_3_PO_4_ in water (*v*/*v*), methanol, and acetonitrile (96/2/2 (*v*/*v*/*v*)) (gradient elution). A preliminary empty gradient chromatographic run was performed to make sure that there were no interfering coelution peaks by injecting 20 µL of methanol/water 80/20 (*v*/*v*) into the HPLC system. Furthermore, 20 µL of the external calibration standard solution (tyrosol and syringic acid) was injected. The values of the response factors (RF) for 1 µg of tyrosol and 1 µg of syringic acid were calculated (called RRFsyr/tyr). After that, 20 µL of the final sample solution was injected into the HPLC system, and the chromatogram at 280 nm was recorded. Polyphenol content, expressed in mg/kg, was calculated by summing the areas of the different chromatographic peaks using the following formula, expressing the result with two decimals:

(5) $$Polyphenols\;content\;\left(\frac{mg}{kg}\right)=\frac{\left(\Sigma A\right)\times 1.000\times \frac{RRFsyr}{tyr}\times \left(W\;syringic\;acid\right)}{\left(A\;syringic\;acid\right)\times \left(W\right)}\;$$

where ΣA is the sum of the areas of the peaks of the polyphenols (hydroxytyrosol, tyrosol, natural and oxidized derivatives of oleuropein and ligstroside, lignans, flavonoids, and phenolic acids) recorded at 280 nm; A syringic acid: is the area of the internal standard (syringic acid) recorded at 280 nm; 1.000 is the factor used to express the result in mg/kg; W is the weight in grams of the oil sample used; RRFsyr/tyr is the multiplication coefficient to express the final results in tyrosol; W syringic acid is the weight in milligrams of the syringic acid used as an internal standard in 1 mL of the solution added to the sample during the extraction.

### 2.8. Sensory Analysis

The official procedures described in Commission Regulation (EEC) No 2568/91 [33], with subsequent amendments [35,36], and the method of the International Olive Council [31] were followed to assess the organoleptic evaluation of the EVOO samples. The panel consisted of a panel leader and 10 trained assessors (5 men and 5 women, aged 25 to 52 years old at the time of the study, with an average age of 37 years). The olfactory intensities were graded using a continuous scale ranging from 0 (no perceived sensation) to 10 (maximum intensity of perceived sensation). The panel was used to assess the intensity and balance of fruity (green or ripe) sensations, bitterness, and pungency. The intensities of the defect attributes were graded using a similar scale, evaluating the intensity and balance of rancidity and other defects). Informed consent was obtained from all individual participants included in the study. The descriptors evaluated included the negative attributes, which indicated the intensity of defects, such as fusty/muddy sediment, musty/humid/earthy, winey/vinegary/acid/sour, frostbitten olives (wet wood), and rancid. Additionally, the panelists evaluated other negative attributes, e.g., metallic, dry hay, grubby, rough, brine, heated or burnt, vegetable water, esparto, cucumber, and greasy, and finally evaluated the positive attributes, such as green fruity, ripe fruity, bitter, and pungency, on a 10 cm unstructured line scale for further calculation and statistical analyses. The results were entered in Microsoft Excel (Office 365, Microsoft Corporation, Redmond, WA, USA), following the statistical method outlined in the IOC Standard T20 Doc. no. 15 [31].

### 2.9. Statistical Analyses

The analyses were conducted in triplicate, and the obtained data were presented as mean ± standard deviation (SD). One-way analysis of variance (ANOVA) was carried out using SPSS software (SPSS version 28, IBM SPSS Statistics, Chicago, IL, USA), with a post hoc Tukey’s honestly significant difference test. *p* < 0.05 was considered statistically significant. Additionally, the mean sensorial scores were plotted in a radar graph and analyzed using principal component analysis (PCA), i.e., scores and loadings (biplot), hierarchical cluster analysis (HCA) and heatmap visualization, which were performed using Origin version 19 (Origin Lab, Northampton, MA, USA).

## 3. Results and Discussion

### 3.1. Physicochemical Characteristics

#### 3.1.1. Acidity

Physicochemical characteristics provide key information on the quality status and oxidation progress of EVOOs [37]. Moreover, the quality indices of the fried edible oils are of interest in assessing oil quality and can be determined by evaluating these parameters [38]. The acidity of the EVOO samples is shown in Table 2. Acidity values as % oleic acid in Con.1, Con.2, and E.1 were recorded as being 0.17, 0.18, and 0.19%, respectively, and, thus, no significant difference (*p* > 0.05) was observed among these samples. Hence, EVOO enriched with HTyr which was deep fried for 3 h at 170 °C (E.1) had a lower acidity value compared to E.13 (non-supplemented EVOO under the same conditions). A continuous increase in acidity was observed for all olive oil samples, particularly EVOOs without HTyr. In addition, the final values of acidity in EVOOs enriched with HTyr ranged between 0.19% and 0.54% from E.1 to E.9, with a significant increase (*p* < 0.05), but remained under the standard values (≤0.8%) during deep frying, in accordance with the labelling category of EVOO. On the other hand, the final values of acidity in EVOOs without HTyr ranged from 0.20% to 0.78% after the same time/temperature interactions (from E.13 to E.21), with a significant increase (*p* < 0.05) in the acidity (Table 2). Therefore, OFE containing HTyr and its derivatives enhanced the antioxidant potential of EVOO and slowed down the degradation process. This effect could be largely attributed to the antioxidant potential and free radical-scavenging power of phenolic compounds and phytochemicals present in olive fruit extract [39].

**Figure 1:**
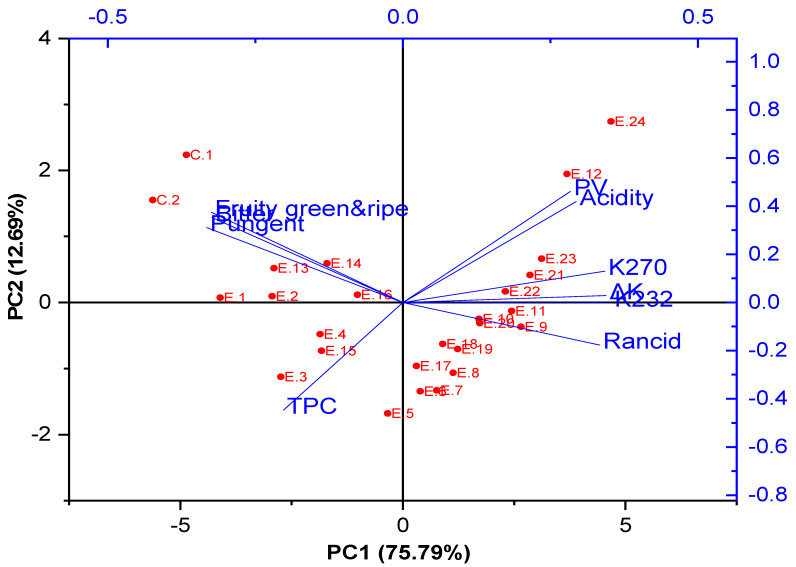
PCA biplot (score and loading plots) of EVOO cv. Manzanilla under different deep frying conditions to illustrate the clustering of samples around the main variables. Refer to Table 2’s caption for the meanings of the oil samples’ abbreviations.

The EVOO acidity gradually increased and exceeded the limits until the end of the experiment (E.10–E.12) and (E.22–E.24) in EVOO enriched with or without HTyr, respectively, due to the hydrolysis of the triglycerides into FFAs at a high temperature for a long time [40], the transformation of the secondary oxidation products to free acids [41], and the loss of the natural antioxidant extract’s ability to inhibit the hydrolysis [42]. In addition, an increase in the acidity was observed in the deep fried oil samples fried for 24 h at 210 °C and above, i.e., E.10, E.11, E.12, E.22, E.23, and E.24, with recorded values of 0.81%, 0.83%, 1.50%, 0.81%, 0.95%, and 1.87%, respectively. These oils, therefore, were not considered EVOOs due to their acidity exceeding the standard limits. This makes sense because long periods of continuous deep frying increase the acidity value due to rancidity, hydrolysis, and degradation of triglycerides [43]. Additionally, PCA analysis showed that E.9, E.10, E.11, E.12, E.20, E.21, E.22, E.23, and E.24 had the highest acidity. The contribution rates of PC1 and PC2 were 75.79% and 12.69%, respectively, collectively explaining 88.46% of the total data variability (Figure 1).

Indeed, acidity levels are directly related to the fatty acids content in oils. While the FFA content is a debated indicator for assessing the degradation of fried oil, it is closely linked to the quality of fried food. FFAs may not affect frying efficiency in practice, but they may have significant unfavorable effects on health or sensory evaluation [44]. Additionally, olive oil is considered to be an EVOO when the acidity value is ≤0.8. Any specific extinction exceeding this value means the oil falls into another olive oil classification according to Commission Regulations EEC 2568 [33].

Therefore, it can be concluded that the incorporation of olive fruit extracts favored the hydrolytic stability of the oils, namely through the control of the acidity of the oils (lower values of oils acidity enriched with extract rather than plain oils). Similarly, the study of Sun-Waterhouse et al. [45], verified that encapsulated and non-encapsulated caffeic acid was effective in slowing down FFA production in EVOO. Likewise, Paulo et al. [46] found that fortifying olive oil with olive mill pomace (OMP)-loaded ethylcellulose microparticles slowed the hydrolysis processes. The microparticles were effectively designed to sustain the release of antioxidants, helping to control the oxidative stability of the oil samples and delay the formation of free fatty acids, in contrast to synthetic antioxidants.

#### 3.1.2. Peroxide Value

The PV is useful in monitoring the initial stage of oxidation, where the primary oxidation products are measured [47], mainly consisting of hydroperoxides that are not stable and decompose, producing secondary oxidation compounds, e.g., aldehydes and ketones that are responsible for an off flavor and sensorial defects [48]. The current findings indicated that Con.2, Con.1, and E.1 revealed the lowest PV values (8.11, 10.09, and 10.65 mEq O_2_/kg, respectively) (Table 2). All olive oils supplemented with HTyr, from E.1 to E.8, as well as olive oils not supplemented with HTyr, from E.13 to E.20, presented close peroxide levels during deep-frying and showed a significant increase (*p* < 0.05) as the frying time increased. The HTyr-EVOO samples exhibited higher peroxide values at 170 °C for 24 h (E.9, with a value of 22.83 mEq O_2_/kg), and in the oil sample heated at 210 °C for 48 h (E.12, with a value of 38.97 mEq O_2_/kg). Additionally, the same trend was observed for non-supplemented oils under the same conditions. Thus, increasing the time of continuous heating led to an increase in the primary oxidation products due to the oxidation of the unsaturated bonds [49].

On the other hand, E.10 (EVOO heated at 210 °C for 24 h) and E.22 (non-supplemented EVOO heated at 210 °C for 24 h) showed a declining trend (14.98 and 20.76 mEq O_2_/kg, respectively), as the PV began to decrease owing to the breaking of the double bond [49]. This decrease can also be explained by the formation of secondary oxidation products, such as hydrocarbons, alcohols, ketones, and aldehydes, from very unstable primary oxidation products, i.e., hydroperoxides. As reported elsewhere, the PV decreases as oxidation proceeds due to the rapid decomposition of hydroperoxides [50].

Additionally, olive oil is considered EVOO when the PV ≤ 20. If any specific extinction exceeds this value, the oil falls into another olive oil classification according to Commission Regulations EEC 2568 [33]. Additionally, the PCA analysis also revealed that E.9, E.10, E.11, E.12, E.20, E.21, E.22, E.23, and E.24 had the highest PV (Figure 1). In summary, EVOO supplemented with olive fruit extract has a lower peroxide value compared to non-supplemented oils under the same deep frying conditions, thanks to the antioxidant potential of hydroxytyrosol and its derivatives. Similarly, Paulo et al. [46] found that fortifying olive oils with OMP-loaded ethylcellulose microparticles delayed the formation of peroxides, thus decreasing the amount of secondary oxidation products. Therefore, polyphenol-rich natural extracts could be a promising strategy to protect against oxidation, offering a better alternative to synthetic, harmful antioxidants. In addition, adding rosemary extract (RosE) to soybean oil, rice bran oil, and cottonseed oil effectively enhanced their protection by boosting the antioxidant capacity and total phenolic content, reducing the peroxide value, and slowing the degradation of tocopherols and polyunsaturated fatty acids [51]. Recently, Singh et al. [52] reported that RosE effectively inhibited the oxidation of polyunsaturated fatty acids, thereby enhancing the oxidative stability of omega-3 fatty acid-rich structured lipids.

#### 3.1.3. Conjugated Dienes and Conjugated Trienes

Hydroperoxides, which indicate the initial stage of oxidation, are characterized by an absorbance maximum at 232 nm [53]. The oxidation of polyunsaturated fatty acids leads to the formation of hydroperoxides. Immediately after the formation of peroxides, the non-conjugated double bonds typically found in natural unsaturated lipids facilitate the regeneration of conjugated dienes (K_232_), which absorb at 232 nm. Measuring K_232_ and K_270_ provides a more reliable assessment of oxidation levels, because these values remain detectable in frying oil [54].

In this regard, conjugated diene (K_232_) is illustrated in Table 2. The results showed that Con. 2 had the lowest K_232_ value (1.48), while Con. 1 exhibited a slightly higher value (1.64). Moreover, there were no significant variations (*p* > 0.05) among E.1, E.2, and E.3. An increment in K_232_ values was observed at the samples treated for 12 h at 170 °C. Additionally, from E.1 to E.7, as well as from E.13 to E.18, the K_232_ values fell within the legal standard values for EVOO. Thus, EVOO is stable during up to 18 h of deep frying at 170 °C in HTyr-EVOOs or during up to 12 h of deep frying at 210 °C in non-HTyr-EVOOs. This indicates that HTyr-EVOOs experienced lower degradation compared to the non-supplemented samples, likely due to the antioxidant potential and free radical scavenging power of the phenolic compounds and phytochemicals contained in OFE [55,56]. On the other hand, the increment in K_232_ was observed when deep frying for a long time (18 h or more), and this may be attributed to the high degradation level and primary oxidation compounds being released from the deep fried oil. Furthermore, PCA analysis revealed that E.9, E.10, E.11, E.12, E.20, E.21, E.22, E.23, and E.24 were clustered around high K_232_ values (Figure 1).

Carbonated compounds and conjugated trienes associated with the secondary oxidation stage result in an increase in the absorbance value at 270 nm. When polyunsaturated fatty acids with three or more double bonds undergo oxidation, the conjugation process leads to the formation of conjugated trienes (K_270_), which absorb at 270 nm. The changes in UV absorbance at 270 nm serve as a relative measure for quantifying oxidation levels [57].

The conjugated triene (K_270_) values are illustrated in Table 2. The results show that Con.1, Con.2, and E.1 recorded the lowest K_270_ values (0.16, 0.17, and 0.21, respectively), and remained within the standard limits (≤0.22). On the other hand, significant variations and increases above the legal limits (*p* < 0.05) in K_270_ values were observed in the samples treated from E.2 to E.24. Consequently, these findings suggest that EVOO is stable for up to 3 h of deep frying at 170 °C with added HTyr, due to its low secondary oxidation compounds. However, the increment was observed when deep frying for a long time or at high temperature, and this was attributed to a high degradation level, and to primary oxidation compounds being converted to secondary oxidation products [58]. In addition, PCA analysis showed that E.9, E.10, E.11, E.12, E.20, E.21, E.22, E.23, and E.24 were clustered around a high K_270_ value (Figure 1).Furthermore, ΔK values are illustrated in Table 2. The purity and degree of degradation of oil can be determined using the ΔK value, which is another main analysis performed for the estimation of EVOO quality. There is a correlation between ΔK and the state of oxidation which can be seen by detecting specific oxidized compounds, some of which are generated from secondary oxidation [59]. The present results indicate that Con. 1, Con. 2, and E.1 exhibited the lowest ΔK values (0.009, 0.008, and 0.010, respectively), all of which remained within the standard limits (≤0.01). However, significant increases (*p* < 0.05), exceeding the legal limits in ΔK values, were observed in the samples from E.2 to E.24. Moreover, PCA analysis also illustrated that E.9, E.10, E.11, E.12, E.20, E.21, E.22, E.23, and E.24 were clustered around a high ΔK value (Table 2). Additionally, olive oil is considered to be EVOO when K_232_ ≤ 2.50, K_270_ ≤ 0.22, and ΔK ≤ 0.01. If any specific factor exceeds these values, the oil falls into another olive oil classification according to Commission Regulations EEC 2568 [33]. Moreover, the incorporation of aqueous olive fruit extracts improved the hydrolytic stability of the EVOO samples, as indicated by lower rancidity levels in oils enriched with OFE compared to plain oils. Similarly, a study by Tsitlakidou et al. [60] demonstrated that incorporating aqueous extracts from post-distillation rosemary residues into emulsion-filled hydrogel beads significantly reduced the oxidation products in encapsulated rapeseed oil.

### 3.2. Evolution of Changes in Phenolic Compounds

#### 3.2.1. Phenolic Alcohols, Lignans, and Flavonoids

Phenolic compounds are the primary bioactive and health-enhancing constituents found in olive oil and the Mediterranean dietary pattern [61]. Consequently, it is essential to monitor the changes in phenolic content during the process of deep frying to evaluate the possible health advantages associated with the use of olive oil. The results in Table 3 show that HTyr (3,4-dihydroxyphenylethanol; 3,4-DHPEA; 4-(2-hydroxyethyl)benzene-1,2-diol) in non-fired Manzanilla original oil (Con.1) recorded 7.33 mg/kg. The initial HTyr content of non-fried EVOO cv. Manzanilla supplemented with OFE (Con.2) was 159.67 mg/kg. Moreover, significant increases (*p* < 0.5) were observed in supplemented EVOOs compared with non-supplemented samples under deep frying, attributable to the supplementation process of EVOO with OFE enriched with HTyr and its derivatives. Notably, HTyr levels were higher (*p* < 0.5) in all supplemented EVOO samples than the initial content in Con. 1 (original non-supplemented EVOO). In addition, the results showed that HTyr is one of the most abundant phenolic fractions, exhibiting thermal stability and maintaining high concentrations for up to 48 h of deep frying at 170 and 210 °C (Table 3). As previously reported, HTyr is widely recognized as a potent antioxidant, free radical scavenger, and regulator of lipid peroxidation in common vegetable oils [62].

Furthermore, tyrosol (Tyr; 2-(4-hydroxyphenyl)-ethanol; p-HPEA) is the second main phenolic compound detected in the supplemented EVOOs and shows high stability compared to other phenolic fractions. In this regard, the results revealed that deep fried supplemented Manzanilla oils (particularly from E.1 to E.9) contained the highest content of Tyr, ranging from 77.09 to 14.53 mg/kg, respectively. These values surpassed those observed in other experiments and non-enriched EVOOs with HTyr (Table 3). Recent studies have highlighted the health benefits of high Tyr and HTyr intake, including reduced arterial inflammation and atherosclerotic lesion microcalcification in healthy older populations [3]. Numerous other investigations have demonstrated that Tyr and HTyr improve oxidative stress and safeguard against LDL peroxidation, which is the first step in the atherosclerosis process. Tyr and HTyr can also efficiently reduce the release of pro-inflammatory cytokines, chemokines, and growth factors, such as IL-6, IL-8, CXCL13, and vascular endothelial growth factor, and counteract molecules capable of engaging inflammatory monocytes and activating macrophages, possibly mitigating the inflammatory response [63,64].

In addition, methyl-luteolin was identified in quantities of 6.10 and 2.46 mg/kg in Con.2 and Con.1, respectively. Luteolin was identified in quantities of 6.75 and 1.57 mg/kg in Con.2 and Con.1, respectively. The contents of luteolin-7-glucoside and apigenin were detected in higher concentrations in non-supplemented EVOOs than in HTyr-EVOOs. However, these flavonoids were degraded rapidly and were absent at the onset of the deep frying process, except in sample E.1, which exhibited stability for luteolin-7-glucoside at 4.49 mg/kg (Table 3). Recent research has demonstrated the potential health benefits of luteolin and its glycosides. These compounds influence glucose metabolism, cellular proliferation, and the apoptotic process, all of which are often disrupted in cancerous cells. This evidence may indicate that luteolin functions as an anticancer agent [65,66].

Interestingly, verbascoside—a water-soluble phenylethanoid glycoside—was detected in high concentration in deep fried supplemented Manzanilla oils. The findings also revealed that verbascoside contents ranged from 54.33 to 16.50 in samples E.1 to E.12, respectively. This phenolic compound demonstrated remarkable stability up to 48 h of continuous deep frying conditions at 210 °C, attributed to the addition of exogenous OFE extract. In contrast, this bioactive component was not detected in any of the non-supplemented EVOOs subjected to deep frying. Verbascoside is a polyphenol glycoside bioactive compound with a wide range of biological effects, including antioxidant, anticancer, antimicrobial, antiviral, and anti-inflammatory powers, and it plays a vital role in the prevention and treatment of various human diseases and disorders [67].

One of the detected lignans in the samples is pinoresinol, which was measured at 7.63 and 51.76 mg/kg in Con.1 and Con.2, respectively. The content ranged from 51.67 to 33.55 mg/kg from E.1 to E.8, respectively. However, in EVOOs deep fried at 170 °C for 24 h (E.9), this content was completely degraded and not detected. Additionally, the same trend was observed for all non-supplemented EVOOs.

In addition, 1-acetoxypinoresinol exhibited higher stability and ranged from 19.73 to 5.60 mg/kg from E.1 to E.24, respectively. 1-acetoxypinoresinol significantly decreased (*p* < 0.05) with increasing time and temperature, with the greatest decrease observed in non-supplemented EVOOs compared to those enriched samples with HTyr. Therefore, 1-acetoxypinoresinol demonstrates greater stability under high thermal conditions compared to other phenolic compounds.

**Figure 2:**
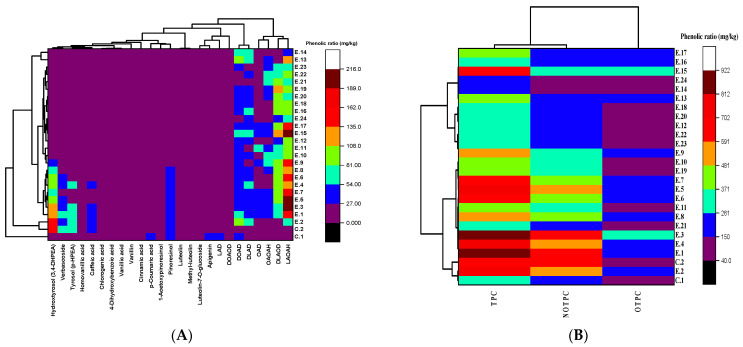
Evolution of phenolic profile of EVOO cv. Manzanilla under different deep frying conditions using hierarchical cluster analysis (HCA) and a heatmap visualization dendrogram. (**A**) Phenolic fractions (mg/kg); (**B**) total phenolic compounds (mg/kg). Where TPC: total phenolic content; NOTPC: non-oxidized total phenolic content; OTPC: oxidized total phenolic content. See Table 2’s caption for the meaning of the oil samples’ abbreviations, and Table 5’s caption for the meaning of the secoiridoid derivatives’ abbreviations.

Therefore, the current findings confirm that HTyr natural extract could be a promising potential food additive and could be added to EVOO not only to enhance its thermal stability but also to extend the health benefits of EVOO by augmenting various phenolic compounds that do not naturally exist or which occur naturally in minimal amounts. Indeed, pinoresinol and 1-acetoxy-pinoresinol have autoprotective, sensory, nutritional, therapeutic, biological, and pharmacological properties. Therefore, this lignan fraction may contribute to the reported beneficial effects that are attributed to polyphenols on human health in a diet rich in olive oil [68]. Consumption of lignans is useful to human health due to their potential anticancer effects, particularly in relation to breast tumors. They also have antifungal and anti-inflammatory aspects [69]. A recent study also reported that 1-acetoxy-pinoresinol exhibited various biological benefits, e.g., anti-diabetic properties through inhibitory activity towards α-glucosidase and α-amylase enzymes, provides protection against neurotoxicity via mediation of Penitrem A toxicity on Schwann cells, exhibits antioxidant activity toward oxidation of liposomes and bulk lipids, and improves the oxidative stability of refined olive oil [70]. Moreover, hierarchical cluster analysis (HCA) and thermograph visualization also showed that HTyr, verbascoside, Tyr, pinoresinol, and 1 acetoxy-pinoresinol were clustered around EVOO samples supplemented with olive fruit extract, notably up to E.8 (deep fried at 210 °C for 18 h with added OFE) (Figure 2A).

#### 3.2.2. Phenolic Acids

The results in Table 4 show the phenolic acid content of supplemented and non-supplemented EVOO cv. Manzanilla with HTyr under deep frying conditions. In this regard, chlorogenic acid was measured at 8.58 mg/kg in Con.2; however, it was not detected in Con.1 (non-supplemented EVOO). Additionally, it exhibited values ranging from 7.38 to 5.05 mg/kg in E.1 to E.4, respectively. Moreover, vanillic acid showed ratios ranging from 5.60 to 5.58 mg/kg in E.1 to E.4, respectively, while 4-dihydroxybenzoic acid varied from 6.82 to 4.38 mg/kg in E.1 to E.4, respectively. On the other hand, these phenolic acids were not detected in samples from E.5 to E.24. Therefore, chlorogenic acid, vanillic acid, and 4-dihydroxybenzoic acid are thermally stable under deep frying conditions up to 210 °C/6 h with added OFE. Consequently, HTyr exogenous natural extract and its derivatives play a significant role (*p* < 0.05) in conserving several pivotal phenolic substances even under high thermal treatments.

*p*-coumaric acid showed ratios ranging from 20.11 to 5.62 mg/kg from E.1 to E.12, respectively. However, it was not detected in all non-supplemented fried EVOO experiments. In addition, cinnamic acid ranged from 17.50 to 6.83 mg/kg from E.1 to E.16, respectively. Thus, these phenolic acids are thermally stable under deep frying conditions up to 210 °C/18 h with added OFE, thanks to the antioxidative and antidegradation power of HTyr and its derivatives. Additionally, the content of *p*-coumaric and cinnamic acids significantly decreased (*p* < 0.05) with the increase in time and temperature (Table 4). Therefore, EVOOs enriched with olive fruit extract could be a more promising frying oil than non-supplemented ones, thanks to their higher content of phenolic acids compared to non-supplemented oils. Indeed, *p*-coumaric acid and its derivatives, such as 4-hydroxycinnamic acid, exhibit a variety of functional properties, including antioxidant, antimicrobial, anticancer, antiarthritic, anti-inflammatory, gout-preventive, anti-diabetic, anti-melanogenic, skin regeneration, gastroprotective, anti-ulcer, cardioprotective, hepatoprotective, reno-protective, bone formation-promoting, anti-angiogenic, and anti-platelet effects, among others [71].

The present results agree with those obtained by De Carvalho et al. [72], who observed that phenolic acids, amongst the phenolic fractions, degrade more quickly than other phenolic compounds. For example, *p*-coumaric acid and ferulic acid were measured at 0.21 and 0.40 mg/kg, respectively, in fresh Brazilian cv. Koroneiki EVOO; however, this content was degraded completely when the oil was deep fried for 1 to 5 days.

**Table 4:** Phenolic acids identified and quantified by HPLC in supplemented (E.1–E.12) and non-supplemented (E.13–E.24) EVOO cv. Manzanilla with HTyr under deep frying conditions compared to non-fried EVOOs (Con.1 and Con.2).

Oil Sample	Chlorogenic Acid	Vanillic Acid	Homovanillic Acid	4-Dihydroxybenzoic Acid	Caffeic Acid	*p*-Coumaric Acid	Cinnamic Acid
Con.1	ND	ND	ND	ND	1.73 ± 0.08 ^d^	36.23 ± 2.48 ^a^	7.04 ± 0.63 ^d^
Con.2	8.58 ± 1.09 ^a^	6.61 ± 0.21 ^a^	20.36 ± 1.61 ^a^	7.86 ± 0.37 ^a^	29.93 ± 1.32 ^a^	14.84 ± 1.87 ^c^	17.50 ± 1.04 ^a^
E.1	7.38 ± 0.85 ^a^	5.60 ± 0.56 ^ab^	19.29 ± 2.57 ^a^	6.82 ± 0.26 ^a^	31.71 ± 2.17 ^a^	20.11 ± 1.12 ^b^	14.26 ± 0.56 ^b^
E.2	6.67 ± 1.13 ^ab^	7.13 ± 0.33 ^a^	13.13 ± 0.86 ^b^	7.22 ± 0.23 ^a^	28.54 ± 1.55 ^a^	18.46 ± 2.09 ^b^	16.41 ± 0.31 ^a^
E.3	6.98 ± 0.58 ^ab^	6.19 ± 0.85 ^ab^	15.80 ± 1.99 ^b^	7.09 ± 0.19 ^a^	27.38 ± 1.13 ^a^	21.44 ± 1.64 ^b^	15.16 ± 1.45 ^b^
E.4	5.05 ± 0.27 ^b^	5.58 ± 0.76 ^ab^	10.23 ± 0.36 ^c^	4.38 ± 0.16 ^b^	27.52 ± 1.77 ^a^	21.97 ± 0.86 ^b^	13.00 ± 0.71 ^b^
E.5	1.05 ± 0.05 ^c^	0.91 ± 0.02 ^c^	8.09 ± 0.42 ^d^	ND	18.33 ± 0.87 ^b^	16.12 ± 0.74 ^c^	9.86 ± 0.19 ^c^
E.6	0.95 ± 0.28 ^c^	0.84 ± 0.08 ^d^	6.03 ± 0.68 ^e^	ND	12.31 ± 0.36 ^c^	14.67 ± 0.58 ^c^	7.25 ± 0.08 ^d^
E.7	0.81 ± 0.07 ^d^	0.71 ± 0.05 ^e^	5.50 ± 0.18 ^e^	ND	18.09 ± 0.29 ^b^	10.94 ± 0.33 ^d^	8.73 ± 0.67 ^c^
E.8	0.21 ± 0.02 ^e^	0.13 ± 0.03 ^f^	3.38 ± 0.64 ^f^	ND	12.06 ± 0.83 ^c^	9.31 ± 1.01 ^d^	7.10 ± 0.15 ^d^
E.9	0.09 ± 0.01 ^f^	0.07 ± 0.01 ^g^	ND	ND	ND	9.40 ± 0.65 ^d^	13.65 ± 0.37 ^b^
E.10	ND	ND	ND	ND	ND	9.79 ± 0.74 ^d^	12.14 ± 0.48 ^b^
E.11	ND	ND	ND	ND	ND	7.40 ± 0.69 ^de^	9.65 ± 0.78 ^c^
E.12	ND	ND	ND	ND	ND	5.62 ± 0.71 ^e^	7.54 ± 0.21 ^d^
E.13	ND	ND	ND	ND	ND	ND	6.42 ± 0.19 ^d^
E.14	ND	ND	ND	ND	ND	ND	5.42 ± 0.27 ^e^
E.15	ND	ND	ND	ND	ND	ND	6.83 ± 0.16 ^d^
E.16	ND	ND	ND	ND	ND	ND	ND
E.17	ND	ND	ND	ND	ND	ND	ND
E.18	ND	ND	ND	ND	ND	ND	ND
E.19	ND	ND	ND	ND	ND	ND	ND
E.20	ND	ND	ND	ND	ND	ND	ND
E.21	ND	ND	ND	ND	ND	ND	ND
E.22	ND	ND	ND	ND	ND	ND	ND
E.23	ND	ND	ND	ND	ND	ND	ND
E.24	ND	ND	ND	ND	ND	ND	ND

^a,b,c,d,e,f^ and ^g^: Data in the same column followed by different superscript letters differ significantly (*p* < 0.05). ND: not detected. Refer to Table 2’s caption for the meaning of the oil samples’ abbreviations.

#### 3.2.3. Secoiridoids and Their Derivatives

Secoiridoids are increasingly recognized as potential therapeutic agents for a range of diseases linked to oxidative stress, demonstrating diverse pharmacological properties, notably including anti-diabetic, antioxidant, anti-inflammatory, immunosuppressive, neuroprotective, anticancer, and anti-obesity activities [73,74]. Oleuropein and ligstroside, the main secoiridoids found in EVOO, are characterized by an elenolic acid core, a glucosidic residue, and a side chain containing either tyrosol (ligstroside) or hydroxytyrosol (oleuropein) attached by an ester linkage. One of the most abundant phenolic components detected in EVOO cv. Manzanilla under deep frying conditions is decarboxymethyl oleuropein aglycone dialdehyde (DOAD), an oleuropein derivative also known as oleacein (3,4-DHPEA-EDA), which is a decarboxymethyl elenolic acid linked to hydroxytyrosol. DOAD was measured at 35.06 mg/kg in Con.1, while it was recorded at 15.80 mg/kg in Con.2. Additionally, DOAD increased by 360% in E.1 compared to the non-fried sample (Con.2) and by 242% in E.13 compared to Con.1. Furthermore, there was a significant increasing trend in DOAD levels (*p* < 0.05) with increasing time and temperature in both supplemented and non-supplemented EVOOs. However, with ongoing increases in time and temperature, the DOAD content decreased significantly (*p* < 0.05) (see Table 5). In addition, two active compounds in virgin olive oil are characterized by a dialdehyde moiety conjugated with 3,4-DHPEA, namely 3,4-DHPEA-EDA (oleacein) and with *p*-HPEA, known as *p*-HPEA-EDA (oleocanthal). These compounds show notable sensory and health benefits. Notably, DOAD (3,4-DHPEA-EDA) contributes to the intense bitterness of EVOO and is associated with several biological activities for human health. The increase in DOAD due to a prolonged deep frying time was also observed in recent research, where a 197.39% increase was reported compared to the original level after one day of continuous deep frying in virgin olive oil [72].

Furthermore, other phenolic compounds were identified, namely DOAOD (decarboxymethyl oleuropein aglycone, oxidized dialdehyde form; oxidized decarboxymethyl 3,4-DHPEA-EDA; oxidized phenolic compound 1). DOAOD is an oxidized isomer of DOAD, with a content of 13.09 mg/kg in Con.1, whereas it was lower in Con.2 (3.25 mg/kg). Upon the initiation of deep frying (170 °C for 3 h), the DOAOD content increased. However, it was not detected at higher temperatures (210 °C) during the same period, and this trend continued up to E.11. From E.13 to E.24, DOAOD was not detected, which could be attributed to its conversion into other oleuropein isomers during intensive heat treatments.

Furthermore, OAD (oleuropein aglycone, dialdehyde form; 3,4-DHPEA-EDA) showed instability during deep frying, likely due to its conversion into other oleuropein isomers because of thermal processes and oxidation. There are limited reports in the literature concerning the oxidized forms of oleuropein aglycone during the deep frying of EVOOs. However, Abbattista et al. [75] reported that a combination of enzymatic and chemical hydrolysis of oleuropein aglycone in virgin olive oil (VOO) leads to dramatic changes, forming multiple oleuropein aglycone isomers. Two groups of isomers with an open structure, previously proposed in the literature and differing only by the location of a C=C bond, exhibit higher polarity. In contrast, closed isomeric forms, characterized by the presence of a dihydropyranic ring, are considered the most stable forms of oleuropein aglycone in VOO.

**Table 5:** Secoiridoid derivatives and total phenolic content (TPC) identified and quantified by HPLC in supplemented (E.1–E.12) and non-supplemented (E.13–E.24) EVOO cv. Manzanilla with HTyr under several deep frying conditions compared to non-fried EVOOs (Con.1 and Con.2).

Oil Sample	DOAD	DOAOD (Oxidized 1)	OAD	DLAOD (Oxidized 2)	LAD	DLAD	OAOAH (Oxidized 3)	LAOAH (Oxidized 4)
Con.1	35.06 ± 2.65 ^f^	13.09 ± 1.08 ^bc^	ND	7.75 ± 0.94 ^g^	49.96 ± 4.55 ^a^	23.29 ± 1.02 ^f^	3.38 ± 0.81 ^h^	8.28 ± 0.74 ^h^
Con.2	15.80 ± 1.45 ^h^	3.25 ± 0.36 ^d^	ND	5.73 ± 0.23 ^g^	15.30 ± 2.29 ^c^	19.22 ± 2.33 ^f^	15.39 ± 2.03 ^fg^	9.47 ± 1.25 ^h^
E.1	54.59 ± 3.16 ^d^	23.56 ± 1.26 ^a^	28.54 ± 2.12 ^cd^	56.41 ± 3.41 ^d^	16.95 ± 1.01 ^c^	51.73 ± 3.91 ^bc^	43.10 ± 3.87 ^c^	140.53 ± 5.34 ^c^
E.2	81.81 ± 2.15 ^a^	0.99 ± 0.02 ^e^	ND	16.04 ± 1.37 ^f^	11.93 ± 2.06 ^d^	73.23 ± 3.56 ^a^	9.11 ± 1.58 ^g^	70.09 ± 3.96 ^i^
E.3	48.17 ± 2.65 ^de^	8.84 ± 0.14 ^d^	31.39 ± 2.65 ^c^	76.15 ± 4.56 ^c^	10.62 ± 0.81 ^d^	45.40 ± 3.88 ^b^	51.84 ± 2.74 ^b^	200.49 ± 9.28 ^a^
E.4	53.01 ± 3.79 ^d^	ND	14.39 ± 1.98 ^ef^	93.31 ± 4.53 ^b^	7.62 ± 0.37 ^e^	54.72 ± 4.52 ^b^	18.41 ± 0.19 ^f^	125.95 ± 7.27 ^d^
E.5	51.16 ± 2.47 ^d^	11.11 ± 0.33 ^c^	36.31 ± 3.22 ^bc^	88.73 ± 2.21 ^b^	ND	43.96 ± 2.91 ^b^	44.29 ± 2.41 ^c^	193.76 ± 8.56 ^b^
E.6	48.33 ± 4.96 ^de^	ND	9.36 ± 0.57 ^f^	99.50 ± 5.59 ^ab^	ND	51.94 ± 1.53 ^b^	23.94 ± 1.96 ^e^	142.13 ± 6.56 ^c^
E.7	50.78 ± 2.32 ^d^	15.94 ± 1.06 ^b^	43.29 ± 3.92 ^b^	85.11 ± 3.01 ^b^	ND	35.88 ± 1.93 ^de^	43.46 ± 3.22 ^c^	170.99 ± 11.21 ^b^
E.8	45.92 ± 1.87 ^f^	0.45 ± 0.03 ^e^	11.70 ± 0.43 ^f^	88.34 ± 2.48 ^b^	ND	43.78 ± 6.45 ^b^	38.73 ± 2.56 ^d^	113.97 ± 8.47 ^d^
E.9	28.45 ± 1.79 ^g^	20.91 ± 0.98 ^a^	48.27 ± 2.02 ^a^	83.57 ± 7.63 ^b^	ND	21.77 ± 4.11 ^f^	66.54 ± 3.82 ^a^	160.66 ± 12.56 ^bc^
E.10	29.63 ± 1.54 ^g^	ND	15.94 ± 0.73 ^ef^	72.92 ± 8.45 ^a^	ND	33.12 ± 3.07 ^de^	65.14 ± 4.51 ^a^	104.37 ± 19.03 ^b^
E.11	32.45 ± 1.19 ^f^	15.94 ± 0.27 ^b^	55.72 ± 4.61 ^a^	73.43 ± 5.99 ^d^	ND	20.78 ± 1.19 ^e^	46.51 ± 2.17 ^c^	90.15 ± 7.31 ^e^
E.12	37.98 ± 2.03 ^f^	ND	15.94 ± 1.69 ^ef^	49.44 ± 6.02 ^c^	ND	32.12 ± 2.96 ^d^	19.22 ± 2.54 ^f^	92.17 ± 4.98 ^f^
E.13	85.34 ± 1.56 ^a^	ND	20.77 ± 1.34 ^e^	23.00 ± 2.81 ^f^	23.03 ± 1.65 ^b^	66.67 ± 1.57 ^a^	40.03 ± 4.42 ^d^	125.49 ± 5.24 ^d^
E.14	73.35 ± 2.74 ^b^	ND	ND	8.61 ± 0.26 ^g^	21.11 ± 2.44 ^b^	72.96 ± 5.06 ^a^	6.30 ± 0.49 ^b^	45.07 ± 2.97 ^g^
E.15	64.93 ± 3.63 ^c^	ND	32.37 ± 2.71 ^d^	111.21 ± 3.48 ^a^	20.66 ± 1.01 ^b^	60.84 ± 2.18 ^b^	51.80 ± 0.53 ^b^	215.35 ± 10.48 ^a^
E.16	37.68 ± 1.28 ^g^	ND	15.47 ± 1.56 ^ef^	83.51 ± 2.54 ^b^	19.35 ± 1.98 ^b^	62.41 ± 2.37 ^b^	11.85 ± 0.74 ^g^	94.85 ± 7.22 ^e^
E.17	49.67 ± 4.17 ^de^	ND	31.90 ± 0.96 ^c^	84.31 ± 4.91 ^b^	18.32 ± 0.73 ^bc^	44.17 ± 4.89 ^c^	41.24 ± 11.86 ^c^	160.70 ± 21.37 ^bc^
E.18	42.92 ± 3.65 ^e^	ND	ND	92.96 ± 3.87 ^b^	19.88 ± 0.32 ^b^	48.74 ± 2.36 ^c^	18.08 ± 6.53 ^b^	88.89 ± 18.56 ^e^
E.19	41.18 ± 5.29 ^e^	ND	26.03 ± 2.35 ^d^	121.48 ± 5.21 ^a^	19.47 ± 1.08 ^ib^	38.20 ± 4.07 ^d^	35.13 ± 11.56 ^d^	89.62 ± 21.51 ^e^
E.20	39.06 ± 4.35 ^e^	ND	ND	82.96 ± 3.04 ^b^	15.12 ± 0.74 ^c^	40.74 ± 2.37 ^d^	28.08 ± 3.56 ^e^	78.89 ± 13.84 ^ef^
E.21	24.84 ± 5.63 ^a^	ND	21.91 ± 4.29 ^e^	87.37 ± 5.21 ^b^	16.88 ± 1.22 ^c^	18.20 ± 3.56 ^a^	55.13 ± 9.69 ^b^	67.85 ± 19.08 ^f^
E.22	26.00 ± 1.09 ^g^	ND	24.82 ± 5.48 ^d^	59.15 ± 2.06 ^d^	5.36 ± 0.31 ^e^	30.74 ± 2.23 ^e^	60.45 ± 4.42 ^a^	86.01 ± 18.57 ^e^
E.23	34.07 ± 5.86 ^f^	ND	17.80 ± 5.72 ^e^	70.66 ± 5.58 ^c^	14.70 ± 0.89 ^c^	23.38 ± 4.94 ^f^	43.92 ± 2.31 ^d^	79.62 ± 22.26 ^ef^
E.24	23.86 ± 1.45 ^g^	ND	19.15 ± 3.11 ^e^	43.59 ± 2.37 ^e^	10.36 ± 0.37 ^d^	34.12 ± 2.43 ^de^	16.64 ± 2.06 ^f^	68.38 ± 14.48 ^f^

^a,b,c,d,e,f,g,h^ and ^i^: Data in the same column followed by different superscript letters differ significantly (*p* < 0.05). DOAD: decarboxymethyl oleuropein aglycone, dialdehyde form; DOAOD: decarboxymethyl oleuropein aglycone, oxidized dialdehyde form (oxidized phenolic compound 1); OAD: oleuropein aglycone, dialdehyde form; DLAOD: decarboxymethyl ligstroside aglycone, oxidized dialdehyde form (oxidized phenolic compound 2); LAD: ligstroside aglycone, dialdehyde form; DLAD: decarboxymethyl ligstroside aglycone, dialdehyde form; OAOAH: oleuropein aglycone, oxidized aldehyde and hydroxylic form (oxidized phenolic compound 3); LAOAH: ligstroside aglycone, oxidized aldehyde and hydroxylic form (oxidized phenolic compound 4). ND: not detected. Refer to Table 2’s caption for the meaning of the oil samples’ abbreviations.

The second main oxidized phenolic compound formed in EVOOs during deep frying is DLOAD. DLOAD, also known as oleocanthal (decarboxymethyl ligstroside aglycone, oxidized dialdehyde form; oxidized p-HPEA-EDA; isomer of ligstroside aglycone; oxidized phenolic compound 2), was measured at 7.75 mg/kg in Con.1, while it was recorded at 5.73 mg/kg in Con.2. Moreover, DLOAD increased by 984% in E.1 compared to the non-fried sample (Con.2), while it increased by 296% in E.13 compared to Con.1. The high DLOAD increase in supplemented EVOOs could be attributed to the elevated tyrosol content, and after initiating the deep frying process, these compounds were changed into tyrosol derivatives. Moreover, there is a significant increasing trend of DLOAD (*p* < 0.05) with the increase in time and temperature in both supplemented EVOOs and non-enriched ones. As the time/temperature interactions gradually increased, the content decreased significantly (*p* < 0.05) at high temperatures and long durations (see Table 5).

The present findings illustrate that LAD (ligstroside aglycone, dialdehyde form; p-HPEA-EDA) was measured at 49.96 mg/kg in Con.1, while it was recorded at 15.30 mg/kg in Con.2. In addition, LAD was not detected in EVOOs enriched with HTyr (from E.5 to E.12) due to the lower content in the original supplemented oil before deep frying, compared to non-supplemented samples before the thermal process. In contrast to other secoiridoid derivatives, LAD exhibited a significant decreasing (*p* < 0.05) trend with the progression of high thermal processing (Table 5).

Additionally, DLAD (decarboxymethyl ligstroside aglycone, dialdehyde form; p-HPEA-EDA; isomer form of ligstroside aglycone) was measured at 23.29 mg/kg in Con.1, while it was detected at 19.22 mg/kg in Con.2. DLAD increased by 269% in E.1 compared to the non-fried sample (Con.2), while it rose by 286% in E.13 compared to Con.1. Moreover, there is a significant increasing trend of DLAD (*p* < 0.05) with the increase in time and temperature in both supplemented EVOO samples and non-enriched ones. However, with the continued increase in time/temperature, the DLAD content reduced significantly (*p* < 0.05). This decrease could be attributed to DLAD being oxidized to DLAOD (oxidized phenolic compound 2) by the prolonged deep frying (Table 5).

OAOAH (oleuropein aglycone, oxidized aldehyde and hydroxylic form; oxidized phenolic compound 3) was recorded at 3.38 mg/kg in Con.1, while it was detected at 15.39 mg/kg in Con.2. Moreover, OAOAH increased by 280% in E.1 compared to the non-fried sample (Con.2), while it rose by 1184% in E.13 compared to Con.1. Moreover, at the beginning of the deep frying, there was a significant increase in OAOAH (*p* < 0.05) with the increase in time and temperature in both supplemented and non-supplemented oil samples. However, with the gradual increase in time/temperature, the OAOAH content decreased significantly (*p* < 0.05). This could be because OAOAH was degraded at the high temperature for a prolonged period (Table 5).

The current results indicated that one of the main detected oxidized ligstroside derivatives in EVOO cv. Manzanilla is LAOAH (ligstroside aglycone, oxidized aldehyde and hydroxylic form; oxidized p-HPEA-EDA; isomer form of ligstroside aglycone; oxidized phenolic compound 4), which was measured at 8.28 mg/kg in Con.1, while it was recorded at 9.47 mg/kg in Con.2. Additionally, LAOAH increased by 1483% in E.1 compared to the non-fried sample (Con.2), while it rose by 1515% in E.13 compared to Con.1. Moreover, there is a significant increasing trend of LAOAH (*p* < 0.05) with the increase in time and temperature in both supplemented EVOO samples and non-enriched ones. However, with the ongoing increase in time/temperature, the LAOAH content decreased significantly (*p* < 0.05). However, this decrease in these samples was still higher than the controls; this is probably due to the high thermal process degrading ligstroside aglycone into unstable oxidized isomers (Table 5).

In summary, the findings from this research serve as a valuable basis for the comprehensive structural analysis of various significant secoiridoids found in EVOO during deep frying, specifically ligstroside aglycone and oleuropein aglycone, for which no substantial evidence supporting the existence of multiple isomers has been reported in the existing literature, particularly during high thermal processing of EVOOs.

#### 3.2.4. Total Phenolic Content (TPC)

**Figure 3:**
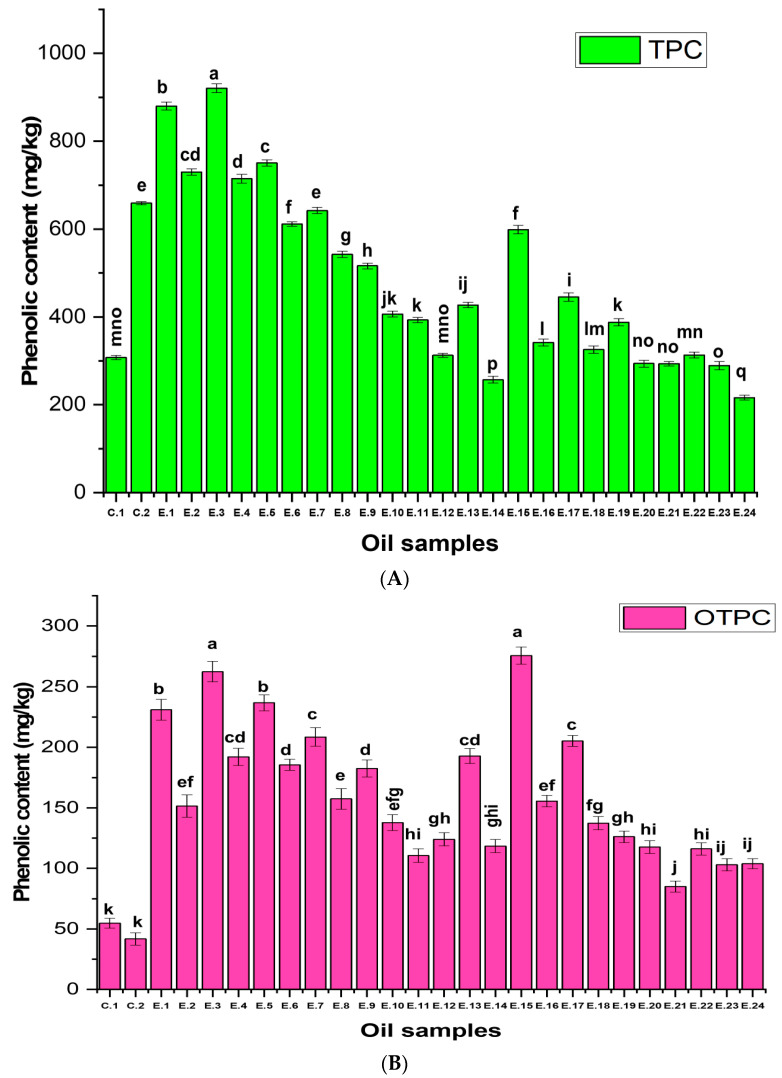

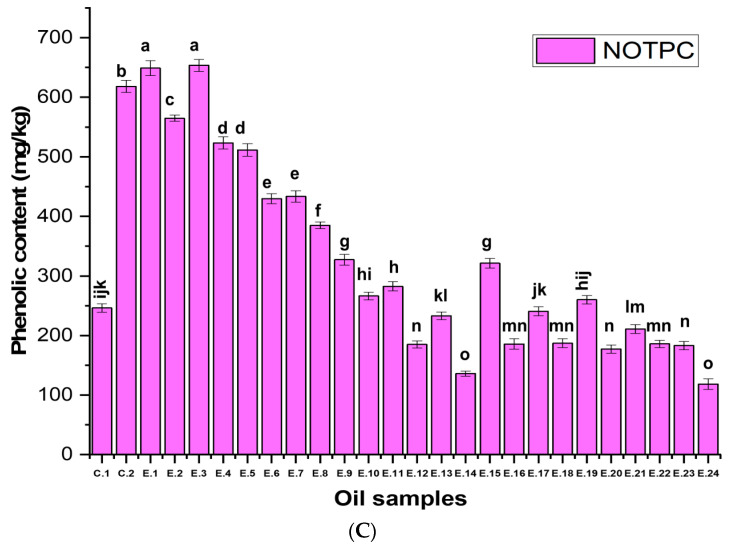
Changes in the TPC (**A**), NOTPC (**B**), and NOTPC (**C**) (mg/kg) values of EVOO cv. Manzanilla under different deep frying conditions, where TPC: total phenolic content, NOTPC: non-oxidized total phenolic content, and OTPC: oxidized total phenolic content. See Table 2’s caption for the meaning of the oil samples’ abbreviations. ^a,b,c,d,e,f,g,h,i,j,k,l,m,n,o,p^ and ^q^: Data followed by different superscript letters among the controls and various experiments are significantly different (*p* < 0.05).

The results in Figure 3A show the changes in TPC, which consists of oxidized total phenolic content (OTPC) and non-oxidized total phenolic content (NOTPC) in EVOO during deep frying. TPC in Con.1 was measured at 307.73 mg/kg, while the initial total phenolic content of supplemented non-fried Manzanilla oil (Con.2) was 659.08 mg/kg. Moreover, E.1 and E.3 exhibited a significantly greater amount of TPC (*p* < 0.05), with values of 879.93 and 920.68 mg/kg, respectively. Additionally, E.2, E.4, and E.5 showed higher TPC than Con.2 (*p* < 0.05). Therefore, TPC increased in these experiments with the onset of the deep frying process, and the same trends were observed for non-supplemented oil samples, which showed a greater TPC (*p* < 0.05) than Con.1.

This increment of TPC in all oils undergoing thermal processing is mainly due to the oxidation process of oleuropein and ligstroside, and consequently, these phenolic fractions are converted to secoiridoid isomers, as shown in the findings from Figure 3B, which demonstrated that a significant increase (*p* < 0.05) in OTPC was observed in all samples, owing to the conversion of secoiridoid derivatives to oxidized forms, e.g., oleuropein and ligstroside isomers. In this regard, E.1 and E.15 presented ratios of 230.96 and 275.52 mg/kg, respectively, and, thus, showed greater OTPC than other treatments (*p* < 0.05), indicating that at the low temperature (170 °C) for 3 or 6 h, OTPC increased (*p* < 0.05). However, by increasing the frying time from 3–48 h at 170 °C or 210 °C, the OTPC exhibited instability and a reduction in these high ratios but was still significantly higher (*p* < 0.05) than the controls, owing to the oxidation phenomenon.

Furthermore, the results of the HCA in Figure 2B illustrated that E.1, E.2, E.4, E.5, E.6, E.7, E.8, E.9, E.13, E.16, and E.17 had higher OTPC contents (ranging from 150–261 mg/kg on the heatmap scale), while E.3 and E.15 exhibited the highest clustering around OTPC, ranging from 261–371 mg/kg on the heatmap scale. Thus, at 170 °C for 6 h of continuous deep frying, the oxidized total phenolic content was recorded at the highest level, and then this content showed a reduction. This is due to the degradation of phenolic substances at high temperatures and over long periods.

One of the most interesting outcomes of the present research is that the TPC in all supplemented EVOOs with OFE was higher (*p* < 0.05) than in the original non-supplemented EVOOs before deep frying, except for E.12 (samples deep fried at 210 °C/48 h with added OFE), which showed no significant difference (*p* > 0.05) compared to Con.1 (Figure 3A). Moreover, PCA analysis also showed that E.1, E.2, E.3, E.4, E.5, E.15, and E.16 were particularly clustered around TPC (Figure 1).

Furthermore, E.6, E.8, E.9, E.10, E.11, and E.12 showed a continuous decrease in their TPC. Therefore, with the progress of time/temperature during deep frying, the phenolic content decreased significantly (*p* < 0.05). The same trend was also observed in E.14, E.20, E.21, E.22, E.23, and E.24. Thus, frying conditions, i.e., time, temperature, and original total polyphenolic content, play a significant role in the thermal oxidative stability of EVOO. In this context, Quiles et al. [76] reported that frying time, type of oils, and the interaction between time and oil affected the TPCs of the oils.

The results of the HCA in Figure 2B illustrate that E.1 and E.3 had higher TPCs (ranging from 812 to 922 mg/kg on the heat map scale), followed by E.2 and E.5, which ranged from 702–812 mg/kg, and then followed by E.4, E.6, and E.7, which exhibited values from 591–702 mg/kg. E.14 and E.24 showed the lowest TPCs (ranging from 150–261 mg/kg). Therefore, EVOO experiments enriched with HTyr and its derivatives had higher phenolic content under continuous and prolonged deep frying. In this regard, another study reported that polyphenolic extract recovered from olive vegetation water (OVW) could reduce oxidation in refined olive oil during a simulated frying process at 180 °C/12 h [77]. Moreover, the current study confirms that the polar phenolic fraction, including HTyr and Tyr, had higher and more stable content under deep frying conditions. On the other hand, non-polar phenolic fractions, namely secoiridoids (e.g., oleuropein and ligstroside), showed instability during deep frying.

Furthermore, the results obtained regarding non-oxidized polyphenols demonstrate that there are significant variations (*p* < 0.05) between the experiments in NOTPC. In this regard, E.1 (deep fried EVOOs at 170 °C/3 h with added HTyr extract) and E.3 (deep fried EVOOs at 170 °C/6 h with added HTyr extract) presented ratios of 648.96 and 653.60 mg/kg, respectively, and, thus, showed higher NOTPC values than other treatments. This indicates that at a low temperature (170 °C) for a short time (i.e., 3 or 6 h), NOTPC remained at a higher level (*p* < 0.05). Following this trend, E.2, E.4, and E.5 exhibited 564.65, 523.34, and 511.38 mg/kg, respectively. However, with the progress of the deep frying, the NOTPC in EVOO samples enriched with OFE decreased significantly (*p* < 0.05) (Figure 3C).

In addition, EVOO experiments supplemented with OFE (from E.1 to E.8) exhibited higher and significantly greater NOTPC (*p* < 0.05) values than all non-supplemented EVOOs, including the original oil before deep frying (Figure 3C). Thus, EVOOs supplemented with OFE could undergo deep frying for up to 18 h at 210 °C without a significant negative impact on the NOTPC values (*p* < 0.05). Furthermore, the results of the HCA in Figure 2B illustrate that E.2, E.4, and E.5 exhibited higher NOTPC contents, ranging from 481–591 mg/kg on the heatmap scale. Meanwhile, Con.2, E.1, and E.3 exhibit the highest clustering around the NOTPC value, ranging from 591–702 mg/kg on the heatmap scale.

### 3.3. Sensory Properties

**Figure 4:**
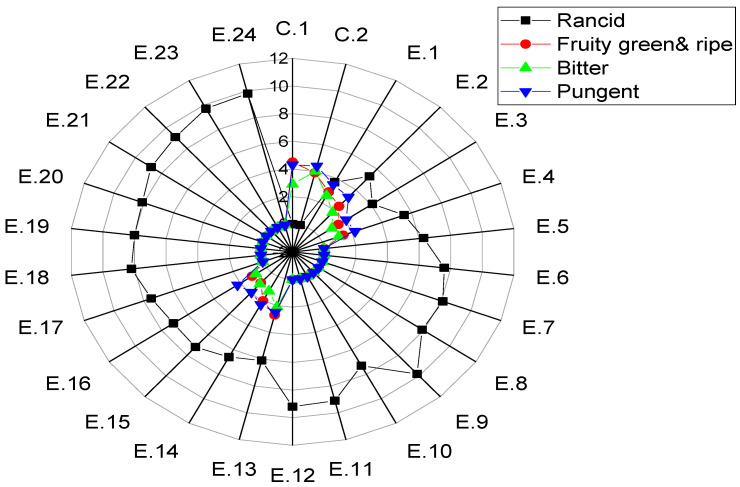
Sensorial qualities of EVOO cv. Manzanilla under different deep frying conditions. See Table 2’s caption for the meaning of the oil samples’ abbreviations.

Among the several organoleptic attributes evaluated by the panelists for different experimental samples of EVOO cv. Manzanilla after deep frying, only five sensory descriptors were successfully developed to characterize the sensory qualities of the samples. These descriptors include one defect, namely rancidity, as well as four positive markers that were perceived, namely fruity (green), fruity (ripe), bitter, and pungent. The findings show that the mean sensory positive scores, i.e., fruity (green), fruity (ripe), bitter, and pungent (Figure 4), significantly decreased during deep frying with increasing time/temperature (*p* < 0.05).

The fruity properties of EVOO experiments enriched with HTyr and its derivatives which underwent thermal processing from 3–6 h at 170–210 °C, i.e., E.1, E.2, E.3, and E.4, were perceived as 2.9, 2.4, 1.5, and 1.4, respectively. On the other hand, non-supplemented samples under the same deep frying conditions were perceived as 2.7, 2.0, 1.0, and 1.1 in E.13, E.14, E.15, and E.16, respectively. Furthermore, E.1, E.2, E.3, and E.4 were perceived as bitter, with values of 2.6, 1.8, 1.0, and 1.1, respectively. In contrast, E.13, E.14, E.15, and E.16 showed values of 2.1, 1.2, 1.1, and 0.8, respectively. Additionally, E.1, E.2, E.3, and E.4 were perceived as being pungent, with values of 3.5, 3.3, 2.1, and 2.2, respectively. In contrast, E.13, E.14, E.15, and E.16 showed values of 2.5, 2.3, 1.9, and 2.2, respectively. Therefore, samples enriched with olive fruit extract at 170–210 °C for up to 6 h showed more desirable organoleptic characteristics than non-supplemented samples. This could be attributed to the higher polyphenol content in the enriched samples, particularly hydroxytyrosol and tyrosol. Positive attributes refer to the distinct flavor profile of a particular olive oil, which encompasses a harmonious blend of green, fruity, bitter, and pungent sensory elements. One of the most crucial factors that affects these attributes is the polyphenolic content.

On the other hand, after deep frying for more than 6 h at 210 °C, sensory positive attributes, e.g., fruity green, fruity ripe, bitterness, and pungency, were reduced to zero. From the obtained findings, it can be summarized that positive sensory attributes might serve as key parameters to differentiate EVOO cv. Manzanilla samples under deep frying conditions into those with highly stable sensorial properties (E.1–E.4) or relatively stable sensory scores (E.13–E.16) from those exhibiting very low sensory attributes after deep frying at 210 °C for between 6 and 48 h.

Furthermore, it appears that even under deep frying conditions, polar polyphenols, i.e., HTyr and Tyr, can be utilized to distinguish high-quality EVOOs from low-quality ones. These compounds also contribute additional sensorial aspects for deep fried EVOOs. Recent reports state that simple phenols, like HTyr and Tyr, have been shown to improve the quality indices and sensorial properties of EVOOs and to extend their shelf life [78,79]. In addition, it has been reported that phenolic compounds are responsible for about 51% of olive oil’s stability, followed by their fatty acid composition (around 24%), in addition to a small contribution from carotenoids and chlorophylls [72,80].

The present findings also showed that E.1, E.2, E.3, and E.4 exhibited low rancidity, with values of 3.7, 5.3, 4.1, and 4.5, respectively. In contrast, E.13, E.14, E.15, and E.16 had higher rancidity values of 6.1, 6.6, 7.2, and 7.1, respectively. Therefore, samples enriched with olive fruit extract demonstrated higher organoleptic desirability than non-supplemented samples. This could be attributed to the higher content of polyphenols, especially hydroxytyrosol and tyrosol, in these samples. In this regard, El Sohaimy et al. [81] studied the correlation between polyphenol content and the sensorial properties of EVOO cv. Manzanilla and cv. Kalamata, which were harvested at various maturity stages. Their findings revealed that high sensory scores, including taste, odor, and overall acceptability, were associated with elevated levels of total phenolic compounds and flavonoids during the early harvesting period. As the TPC gradually decreased, the desirable sensory properties also diminished significantly.

Moreover, the results obtained from the present research indicated that rancidity scores increased significantly (*p* < 0.05) with increasing time/temperature. As illustrated in Figure 4, from E.5 to E.12, the mean rancidity score was ≥6.3, while from E.17 to E.24, the mean rancidity score was ≥7.5. It can be observed that HTyr-EVOOs exhibited lower rancidity compared to non-enriched oil samples under the same conditions.

Furthermore, the higher rancidity could be attributed to chemical oxidation, which promotes the formation of undesirable flavors, such as hept-2-enal and pent-2-enal. These volatile compounds contribute to unpleasant taste profiles, including fusty or muddy sediments, mustiness, and winey, vinegary, or sour notes, as well as rancidity [82].

Furthermore, the low rancidity values in supplemented EVOOs compared to non-enriched ones could be attributed to the high content of HTyr, Tyr, and phenolic acids, which possess notable antioxidant activity. Various recent studies have highlighted that these phenolic compounds have attracted considerable interest due to their biological and pharmacological properties, including their remarkable antioxidant power [83,84].

Moreover, the incorporation of olive fruit extract, which is rich in hydroxytyrosol at a concentration of 650 mg/kg, into the EVOO led to minor degradation and oxidation during the deep frying process. This observation indicates that HTyr and its derivatives play a role in safeguarding fatty acids from oxidation, thereby reducing the hydrolysis of triglycerides. Consequently, the inclusion of the olive fruit hydrolysate extract may delay the onset of oxidation products in the deep fried EVOOs. These findings are consistent with the research conducted by Aydeniz and Yilmaz [85], which validated the influence of phenolic compounds on the acidity levels of frying refining oils. Similarly, the results align with the findings of Zribi et al. [86], who demonstrated that the enrichment of the refined olive oils and refined soybean oils with a hydrolysate olive leaf rich in hydroxytyrosol (500 ppm) resulted in a slight decrease in acidity during pan frying.

The PCA analysis showed that Con.1 and Con.2 were clustered around the fruity attribute (Figure 1). Moreover, E.1, E.2, E.3, E.4, E.13, E.14, E.15, and E.16 separated close to the fruity properties. Regarding bitterness, PCA analysis also revealed that Con.1 and Con.2 clustered around the bitter attribute (Figure 1). Similarly, E.1, E.2, E.3, E.4, E.13, E.14, E.15, and E.16 exhibited higher and more stable bitterness properties, with a similar observation for the pungent attribute. On the other hand, E.6, E.7, E.8, E.9, E.10, E.11, E.12, E.17, E.18, E.19, E.20, E.21, E.22, E.23, and E.24 clustered around rancidity. This was attributed to higher PV, acidity, K_232_, K_270_, and thermo-oxidation as a result of prolonged time and elevated temperature during deep frying. In conclusion, EVOO is thermally stable and demonstrates low rancidity up to 6 h at 210 °C during deep frying. Additionally, the low rancidity observed in EVOOs enriched with HTyr may be attributed to the antioxidant power of the exogenous OFE. Recent research by Esposto et al. [77] reported that phenolic extract recovered from olive vegetation water could reduce oxidation in refined olive oil during a simulated frying process at 180 °C/12 h. Furthermore, olive extract plays a vital role in limiting the production of off-volatile compounds, such as acrolein and β,β-dimethylacrolein. In summary, natural antioxidants are a promising strategy to improve the positive sensorial attributes of edible oils. Furthermore, a recent report by Xi et al. [87] indicated that RosE could enhance the green and fruity aroma and delay the deterioration of walnut oil flavor.

## 4. Conclusions

The present findings indicate that there are notable differences in all evaluated physicochemical parameters across the various experiments. In this regard, HTyr-EVOOs exhibited low degradation progress. Moreover, olive fruit extract (OFE) provided EVOOs with high phenolic content, including simple phenolics, lignans, and phenolic acids even under deep frying conditions. Hydroxytyrosol (HTyr) and tyrosol (Tyr) were among the most stable polyphenols compared to oleuropein and ligstroside, while oxidized secoiridoid derivatives increased significantly with DF progress. The findings also show that prolonged continuous DF of EVOO negatively impacts its total phenolic content. Additionally, EVOO demonstrated thermal stability and had low rancidity up to 6 h at 170 and 210 °C, particularly in HTyr-enriched EVOO samples. However, prolonged thermal processing leads to alterations, including a diminishing of mean positive sensory characteristics and a higher rancidity score. Therefore, the present research identifies the most suitable time/temperature intervals for EVOOs under DF to achieve stable oil quality by maintaining oil stability, particularly by reducing low median defects, such as rancidity, while promoting high and stable positive sensory attributes. Based on these results, it is suggested that natural olive fruit extracts could serve as a promising food additive in deep fried EVOO, to enhance the sensory properties, extend the health benefits through the polyphenolic content, and provide a valuable solution for the food industry by improving the oil’s stability and usability. Further studies are highly recommended to investigate various EVOO categories and/or oils from different origins.

## Figures and Tables

**Table 1:** Experimental design methodology of EVOO cv. Manzanilla supplemented with OFE under different deep frying conditions to illustrate the effect of the independent variables (time, temperature, and polyphenol addition) on the dependent variables (response).

Experiment (E.)	Independent Variables			Response
	Time (h)	Temperature (°C)	Polyphenol Addition (mg/kg)	
Con.1 *	**-**	**-**	302.40	Acidity (%) Peroxide value (meq O_2_/kg) K_232_ K_270_ ΔK Phenolic compounds (mg/kg) Sensory properties
Con.2 **	**-**	**-**	659.42	
E.1	3	170	659.42	
E.2	3	210	659.42	
E.3	6	170	659.42	
E.4	6	210	659.42	
E.5	12	170	659.42	
E.6	12	210	659.42	
E.7	18	170	659.42	
E.8	18	210	659.42	
E.9	24	170	659.42	
E.10	24	210	659.42	
E.11	48	170	659.42	
E.12	48	210	659.42	
E.13	3	170	302.40	
E.14	3	210	302.40	
E.15	6	170	302.40	
E.16	6	210	302.40	
E.17	12	170	302.40	
E.18	12	210	302.40	
E.19	18	170	302.40	
E.20	18	210	302.40	
E.21	24	170	302.40	
E.22	24	210	302.40	
E.23	48	170	302.40	
E.24	48	210	302.40	

*: Con.1: Refers to the original, non-deep fried EVOO, which is not applicable to the supplementation process with polyphenols. This control applies to E.13–E.24. **: Con.2: Refers to the original supplemented, non-deep fried EVOO, which is applicable to the supplementation process with polyphenols. This control applies to E.1–E.12.

**Table 2:** Evaluation of the changes in the physicochemical properties, i.e., the acidity, peroxide value, and spectrophotometric characteristics, of supplemented (E.1–E.12) and non-supplemented (E.13–E.24) EVOO cv. Manzanilla exposed to prolonged deep frying conditions compared to non-fried EVOOs (Con.1 and Con.2).

Experiment	Acidity (% as Oleic Acid)	PV (mEqO_2_/kg)	K_232_	K_270_	ΔK
Con.1	0.17 ± 0.01 ^l^	10.09 ± 0.19 ^k^	1.64 ± 0.25 ^k^	0.16 ± 0.01 ^n^	0.009 ± 0.001 ^j^
Con.2	0.18 ± 0.01 ^l^	8.11 ± 0.21 ^l^	1.48 ± 0.13 ^l^	0.17 ± 0.02 ^n^	0.008 ± 0.001 ^j^
E.1	0.19 ± 0.01 ^l^	10.65 ± 0.15 ^k^	1.79 ± 0.04 ^j^	0.21 ± 0.02 ^m^	0.010 ± 0.001 ^i^
E.2	0.29 ± 0.02 ^j^	11.01 ± 0.22 ^j^	1.82 ± 0.08 ^j^	0.39 ± 0.01 ^k^	0.015 ± 0.002 ^h^
E.3	0.24 ± 0.03 ^k^	10.98 ± 0.19 ^j^	1.81 ± 0.11 ^j^	0.23 ± 0.01 ^l^	0.012 ± 0.001 ^h^
E.4	0.34 ± 0.06 ^i^	12.12 ± 0.28 ^i^	1.86 ± 0.17 ^hj^	0.44 ± 0.05 ^k^	0.021 ± 0.003 ^f^
E.5	0.42 ± 0.01 ^g^	12.05 ± 0.13 ^hi^	2.14 ± 0.05 ^g^	0.49 ± 0.02 ^h^	0.023 ± 0.001 ^e^
E.6	0.44 ± 0.05 ^g^	13.39 ± 0.18 ^g^	2.33 ± 0.18 ^e^	0.54 ± 0.04 ^f^	0.031 ± 0.002 ^d^
E.7	0.49 ± 0.08 ^fg^	13.69 ± 0.24 ^g^	2.41 ± 0.07 ^d^	0.61 ± 0.05 ^e^	0.039 ± 0.001 ^cd^
E.8	0.45 ± 0.04 ^g^	14.11 ± 0.38 ^f^	2.56 ± 0.20 ^c^	0.75 ± 0.02 ^d^	0.041 ± 0.005 ^c^
E.9	0.54 ± 0.01 ^f^	22.83 ± 0.18 ^d^	2.71 ± 0.15 ^b^	1.10 ± 0.18 ^a^	0.040 ± 0.002 ^c^
E.10	0.81 ± 0.03 ^d^	14.98 ± 0.31 ^f^	2.69 ± 0.09 ^b^	0.84 ± 0.05 ^c^	0.042 ± 0.002 ^c^
E.11	0.83 ± 0.04 ^e^	21.18 ± 0.52 ^e^	2.75 ± 0.12 ^b^	0.96 ± 0.01 ^b^	0.054 ± 0.003 ^a^
E.12	1.50 ± 0.09 ^b^	38.97 ± 0.14 ^b^	2.79 ± 0.07 ^ab^	0.99 ± 0.09 ^b^	0.046 ± 0.001 ^bc^
E.13	0.20 ± 0.01 ^lm^	11.02 ± 0.16 ^j^	1.82 ± 0.16 ^j^	0.22 ± 0.01 ^lm^	0.011 ± 0.003 ^i^
E.14	0.32 ± 0.02 ^i^	11.25 ± 0.54 ^i^	1.87 ± 0.21 ^i^	0.43 ± 0.06 ^k^	0.016 ± 0.001 ^g^
E.15	0.27 ± 0.03 ^j^	13.19 ± 0.12 ^g^	1.88 ± 0.08 ^i^	0.26 ± 0.04 ^l^	0.018 ± 0.005 ^g^
E.16	0.36 ± 0.02 ^h^	12.55 ± 0.29 ^h^	1.95 ± 0.18 ^h^	0.48 ± 0.12 ^i^	0.023 ± 0.002 ^f^
E.17	0.47 ± 0.01 ^f^	13.27 ± 0.34 ^g^	2.21 ± 0.31 ^f^	0.52 ± 0.05 ^g^	0.029 ± 0.004 ^d^
E.18	0.49 ± 0.07 ^f^	14.51 ± 0.18 ^f^	2.38 ± 0.22 ^d^	0.56 ± 0.10 ^f^	0.032 ± 0.001 ^d^
E.19	0.52 ± 0.02 ^f^	14.01 ± 0.65 ^f^	2.55 ± 0.15 ^c^	0.63 ± 0.04 ^e^	0.042 ± 0.002 ^c^
E.20	0.61 ± 0.05 ^e^	14.84 ± 0.71 ^f^	2.76 ± 0.11 ^b^	0.77 ± 0.09 ^d^	0.041 ± 0.001 ^c^
E.21	0.78 ± 0.02 ^d^	24.32 ± 0.15 ^d^	2.78 ± 0.08 ^b^	1.20 ± 0.08 ^a^	0.050 ± 0.003 ^b^
E.22	0.81 ± 0.03 ^d^	20.76 ± 0.69 ^e^	2.75 ± 0.14 ^b^	0.84 ± 0.14 ^c^	0.043 ± 0.002 ^c^
E.23	0.95 ± 0.05 ^c^	25.65 ± 0.78 ^c^	2.81 ± 0.18 ^a^	0.98 ± 0.05 ^b^	0.055 ± 0.002 ^a^
E.24	1.87 ± 0.08 ^a^	41.97 ± 0.28 ^a^	2.86 ± 0.09 ^a^	1.16 ± 0.07 ^a^	0.056 ± 0.004 ^a^

^a,b,c,d,e,f,g,h,i,j,k,l,m,^ and ^n^: Data in the same column followed by different superscript letters differ significantly (*p* < 0.05). Where Con.1 represents the non-fried and non-supplemented original EVOO cv. Manzanilla, Con.2 represents the non-fried and supplemented EVOO cv. Manzanilla mixed with original EVOO cv. Manzanilla up to 659.42 mg/kg of polyphenols, E.1: Manzanilla oil deep fried at 170 °C for 3 h with polyphenol supplementation, E.2: Manzanilla oil deep fried at 210 °C for 3 h with polyphenol supplementation, E.3: Manzanilla oil deep fried at 170 °C for 6 h with polyphenol supplementation, E.4: Manzanilla oil deep fried at 210 °C for 6 h with polyphenol supplementation, E.5: manzanilla oil deep fried at 170 °C for 12 h with polyphenol supplementation, E.6: Manzanilla oil deep fried at 210 °C for 12 h with polyphenol supplementation, E.7: Manzanilla oil deep fried at 170 °C for 18 h with polyphenol supplementation, E.8: Manzanilla oil deep fried at 210 °C for 18 h with polyphenol supplementation. E.9: Manzanilla oil deep fried at 170 °C for 24 h with polyphenol supplementation, E.10: Manzanilla oil deep fried at 210 °C for 24 h with polyphenol supplementation. E.11: Manzanilla oil deep fried at 170 °C for 48 h with polyphenol supplementation, E.12: Manzanilla oil deep fried at 210 °C for 48 h with polyphenol supplementation. E.13: Manzanilla oil deep fried at 170 °C for 3 h without polyphenol supplementation, E.14: Manzanilla oil deep fried at 210 °C for 3 h without polyphenol supplementation, E.15: Manzanilla oil deep fried at 170 °C for 6 h without polyphenol supplementation, E.16: Manzanilla oil deep fried at 210 °C for 6 h without polyphenol supplementation, E.17: Manzanilla oil deep fried at 170 °C for 12 h without polyphenol supplementation, E.18: Manzanilla oil deep fried at 210 °C for 12 h without polyphenol supplementation, E.19: Manzanilla oil deep fried at 170 °C for 18 h without polyphenol supplementation, E.20: Manzanilla oil deep fried at 210 °C for 18 h without polyphenol supplementation. E.21: Manzanilla oil deep fried at 170 °C for 24 h without polyphenol supplementation, E.22: Manzanilla oil deep fried at 210 °C for 24 h without polyphenol supplementation. E.23: Manzanilla oil deep fried at 170 °C for 48 h without polyphenol supplementation, and E.24: Manzanilla oil deep fried at 210 °C for 48 h without polyphenol supplementation.

**Table 3:** Selected identified and quantified phenolic compounds, i.e., phenolic alcohols, phenolic aldehydes, polyphenol glucosides, flavonoids, and lignans by HPLC of supplemented (E.1–E.12) and non-supplemented (E.13–E.24) EVOO cv. Manzanilla with HTyr under several deep frying conditions compared to non-fried EVOOs (Con.1 and Con.2).

Oil Sample	Phenolic Alcohols		Phenolic Aldehydes	Polyphenol Glycoside	Flavonoids				Lignans	
	HTyr	Tyr	Vanillin	Verbascoside	Luteolin-7-Glucoside	Luteolin	Apigenin	Methyl-Luteolin	Pinoresinol	1-Acetoxypinoresinol
Con.1	7.33 ± 0.21 ^h^	9.36 ± 1.03 ^e^	3.90 ± 0.65 ^c^	2.21 ± 0.04 ^l^	10.70 ± 1.82 ^a^	1.57 ± 0.21 ^b^	27.85 ± 2.96 ^a^	2.46 ± 0.18 ^b^	37.63 ± 1.07 ^d^	14.67 ± 0.23 ^b^
Con.2	159.67 ± 3.09 ^a^	78.04 ± 4.51 ^a^	10.49 ± 1.02 ^a^	67.14 ± 3.61 ^a^	4.47 ± 0.71 ^b^	6.75 ± 0.46 ^a^	22.66 ± 0.17 ^b^	6.10 ± 0.74 ^a^	51.76 ± 3.65 ^a^	17.88 ± 0.66 ^a^
E.1	137.22 ± 4.01 ^b^	77.09 ± 1.79 ^a^	10.18 ± 0.83 ^a^	54.33 ± 2.76 ^a^	4.49 ± 0.22 ^b^	ND	ND	ND	51.67 ± 1.81 ^a^	19.73 ± 1.42 ^a^
E.2	138.43 ± 2.63 ^b^	77.99 ± 2.43 ^a^	6.47 ± 0.62 ^b^	51.96 ± 3.97 ^ab^	ND	ND	ND	ND	50.23 ± 3.36 ^a^	20.97 ± 0.86 ^a^
E.3	114.48 ± 4.91 ^c^	75.27 ± 1.47 ^a^	10.77 ± 0.96 ^a^	45.91 ± 1.22 ^b^	ND	ND	ND	ND	48.74 ± 3.07 ^a^	19.63 ± 1.34 ^a^
E.4	90.05 ± 3.45 ^d^	57.34 ± 0.89 ^b^	ND	33.34 ± 3.56 ^c^	ND	ND	ND	ND	39.61 ± 1.13 ^b^	20.10 ± 0.63 ^a^
E.5	95.53 ± 1.51 ^d^	26.28 ± 1.04 ^c^	ND	41.76 ± 2.91 ^b^	ND	ND	ND	ND	44.29 ± 0.98 ^a^	17.27 ± 0.21 ^a^
E.6	91.82 ± 3.08 ^d^	24.81 ± 0.72 ^c^	ND	27.05 ± 0.83 ^cd^	ND	ND	ND	ND	35.02 ± 1.38 ^b^	13.44 ± 0.78 ^b^
E.7	69.44 ± 2.01 ^e^	23.46 ± 2.51 ^c^	ND	23.82 ± 2.74 ^d^	ND	ND	ND	ND	38.75 ± 2.24 ^b^	12.08 ± 1.12 ^b^
E.8	64.68 ± 0.01 ^e^	21.78 ± 1.33 ^c^	ND	21.45 ± 1.06 ^d^	ND	ND	ND	ND	33.55 ± 1.67 ^bc^	10.64 ± 0.36 ^b^
E.9	27.49 ± 0.77 ^f^	14.53 ± 0.28 ^d^	ND	21.85 ± 0.73 ^d^	ND	ND	ND	ND	ND	12.79 ± 0.65 ^b^
E.10	20.28 ± 1.03 ^g^	10.96 ± 0.91 ^e^	ND	20.67 ± 2.96 ^d^	ND	ND	ND	ND	ND	9.15 ± 0.13 ^c^
E.11	19.30 ± 1.11 ^g^	8.52 ± 1.74 ^e^	ND	19.85 ± 0.84 ^f^	ND	ND	ND	ND	ND	10.79 ± 0.78 ^b^
E.12	18.11 ± 2.31 ^g^	6.79 ± 0.93 ^ef^	ND	16.50 ± 1.08 ^f^	ND	ND	ND	ND	ND	7.90 ± 0.36 ^c^
E.13	4.02 ± 0.37 ^i^	9.49 ± 0.71 ^e^	ND	ND	ND	ND	ND	ND	ND	9.13 ± 0.08 ^bc^
E.14	4.34 ± 0.48 ^i^	11.10 ± 1.69 ^e^	ND	ND	ND	ND	ND	ND	ND	4.94 ± 0.73 ^a^
E.15	3.74 ± 0.96 ^i^	8.45 ± 0.81 ^e^	ND	ND	ND	ND	ND	ND	ND	8.89 ± 1.07 ^bc^
E.16	3.14 ± 0.71 ^i^	9.39 ± 0.43 ^e^	ND	ND	ND	ND	ND	ND	ND	5.76 ± 0.26 ^d^
E.17	2.67 ± 0.27 ^ij^	7.47 ± 1.22 ^e^	ND	ND	ND	ND	ND	ND	ND	5.35 ± 0.73 ^d^
E.18	2.00 ± 0.09 ^ij^	7.58 ± 0.85 ^e^	ND	ND	ND	ND	ND	ND	ND	5.60 ± 0.68 ^d^
E.19	2.83 ± 0.11 ^ij^	6.90 ± 0.61 ^ef^	ND	ND	ND	ND	ND	ND	ND	8.27 ± 0.15 ^bc^
E.20	1.29 ± 0.24 ^j^	5.20 ± 0.26 ^f^	ND	ND	ND	ND	ND	ND	ND	5.60 ± 0.39 ^d^
E.21	ND	ND	ND	ND	ND	ND	ND	ND	ND	6.72 ± 0.71 ^d^
E.22	ND	ND	ND	ND	ND	ND	ND	ND	ND	4.32 ± 0.18 ^d^
E.23	ND	ND	ND	ND	ND	ND	ND	ND	ND	5.68 ± 0.64 ^d^
E.24	ND	ND	ND	ND	ND	ND	ND	ND	ND	5.60 ± 0.53 ^d^

^a,b,c,d,e,f,g,h,i,j^ and ^l^: Data in the same column followed by different superscript letters differ significantly (*p* < 0.05). ND: not detected. Refer to Table 2’s caption for the meaning of the oil samples’ abbreviations.

## Data Availability

The original contributions presented in the study are included in the article/Supplementary Material; further inquiries can be directed to the corresponding author.

## Supplementary Materials

The following supporting information can be downloaded at https://www.mdpi.com/article/10.3390/foods14020260/s1, Figure S1. The fortification process of EVOO with OFE enriched with hydroxytyrosol and its derivatives. HTyr: hydroxytyrosol; EVOO: extra virgin olive oil cv. Manzanilla; W: water; O: oil; Figure S2: Tyrosol calibration curve used for the validation of the HPLC method; Figures S3–S28: Each figure presents the results displaying the total ion chromatograms (TICs) from the HPLC analysis of phenolic compounds recorded at 280 nm for each investigated oil experiment under various prolonged deep frying conditions, along with the two control olive oil samples. IA: The intensity of absorbance, mAU: milli-absorbance units, and tR: retention time. Detailed captions for each figure are provided in the Supplementary Materials file of this article.
