# Supplementary material for: Improving the Biostability of Extra Virgin Olive Oil with Olive Fruit Extract During Prolonged Deep Frying
Source: Foods. 2025 Jan 15;14(2):260. doi: 10.3390/foods14020260 (PMC11765049; doi:10.3390/foods14020260)
Supplement: Supplementary file 1 [file foods-14-00260-s001.zip › foods-3409194-supplementary.pdf]

Supplementary materials for the article:

Improving the Biostability of Extra Virgin Olive Oil with Olive Fruit Extract During Prolonged Deep Frying

Taha Mehany, José M. González-Sáiz, and Consuelo Pizarro *

Department of Chemistry, University of La Rioja, 26006 Logroño, Spain; taha.abdellatif@unirioja.es (T.M.); josemaria.gonzalez@unirioja.es (J.M.G.-S.)

* Correspondence: consuelo.pizarro@unirioja.es; Tel.: +34-941299626

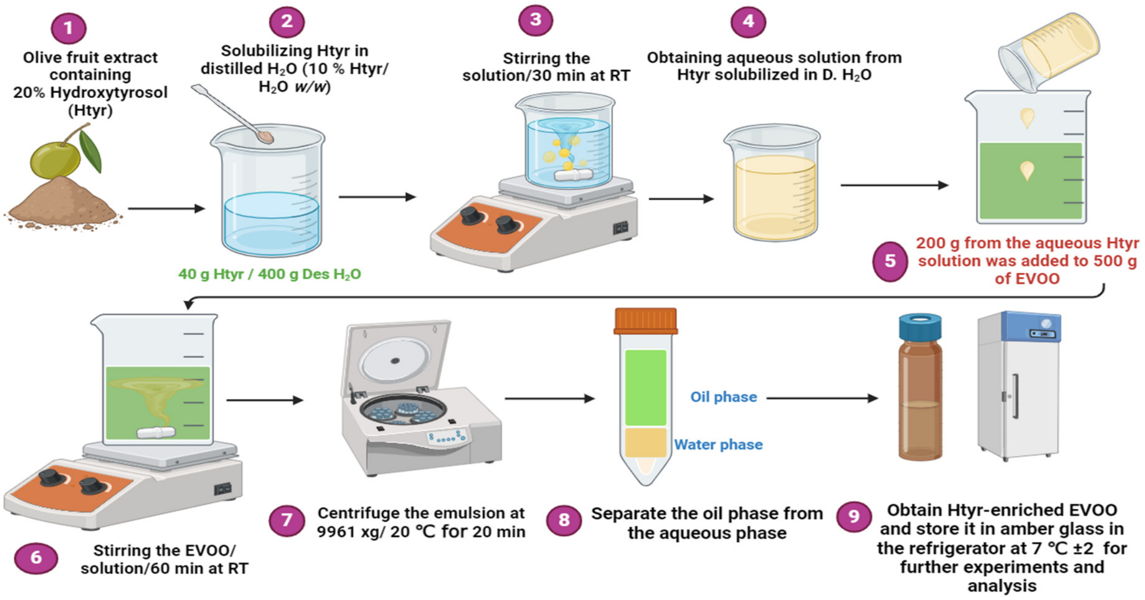

Figure S1. The fortification process of EVOO with OFE enriched with hydroxytyrosol and its derivatives. where: HTyr: hydroxytyrosol; EVOO: extra virgin olive oil cv. Manzanilla; W: water; O: oil.

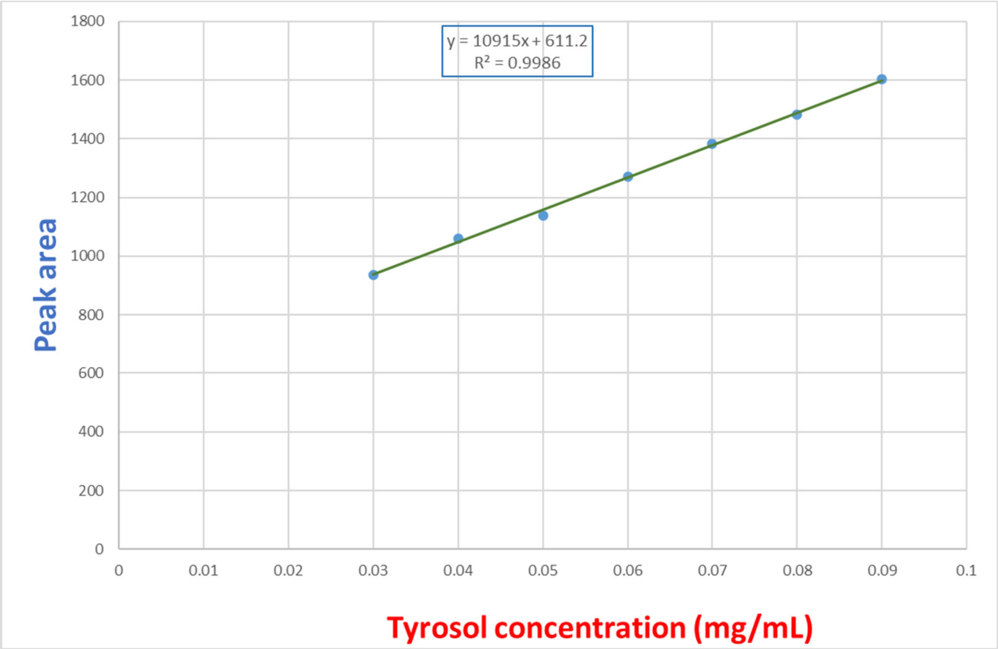

Figure S2. Tyrosol calibration curve used for the validation of the HPLC method.

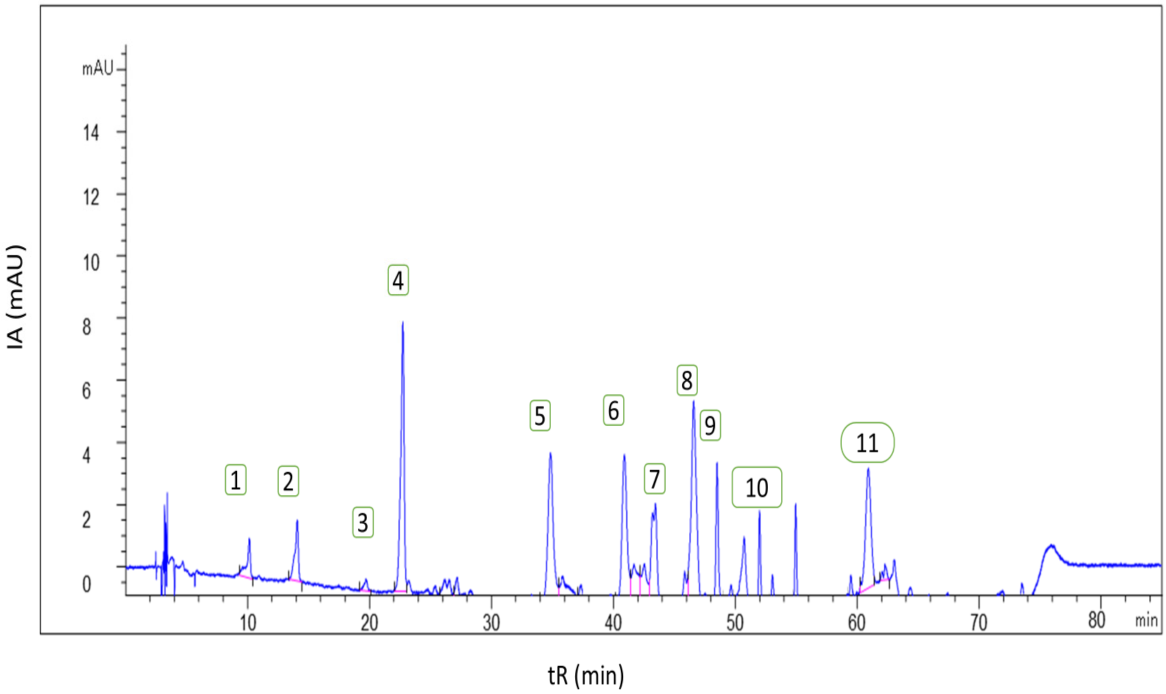

Figure S3. Total ion chromatograms (TICs) from the HPLC analysis of the phenolic compounds recorded at 280 nm in Control 1 (non-deep-fried and original EVOO cv. Manzanilla). (1: Hydroxytyrosol; 2: Tyrosol; 3: caffeic acid; 4: Internal standard (syringic acid); 5: *p*-Coumaric acid; 6: DOAD (Decarboxymethyl oleuropein aglycone, dialdehyde form); 7: DLAD (Decarboxymethyl ligstroside aglycone, dialdehyde form); 8: LAD (Ligstroside aglycone, dialdehyde form); 9: Pinoresinol; 10: 1-Acetoxypinoresinol; and 11: Apigenin).

where: IA: The intensity of absorbance, mAU: milli-Absorbance Units, tR: retention time.

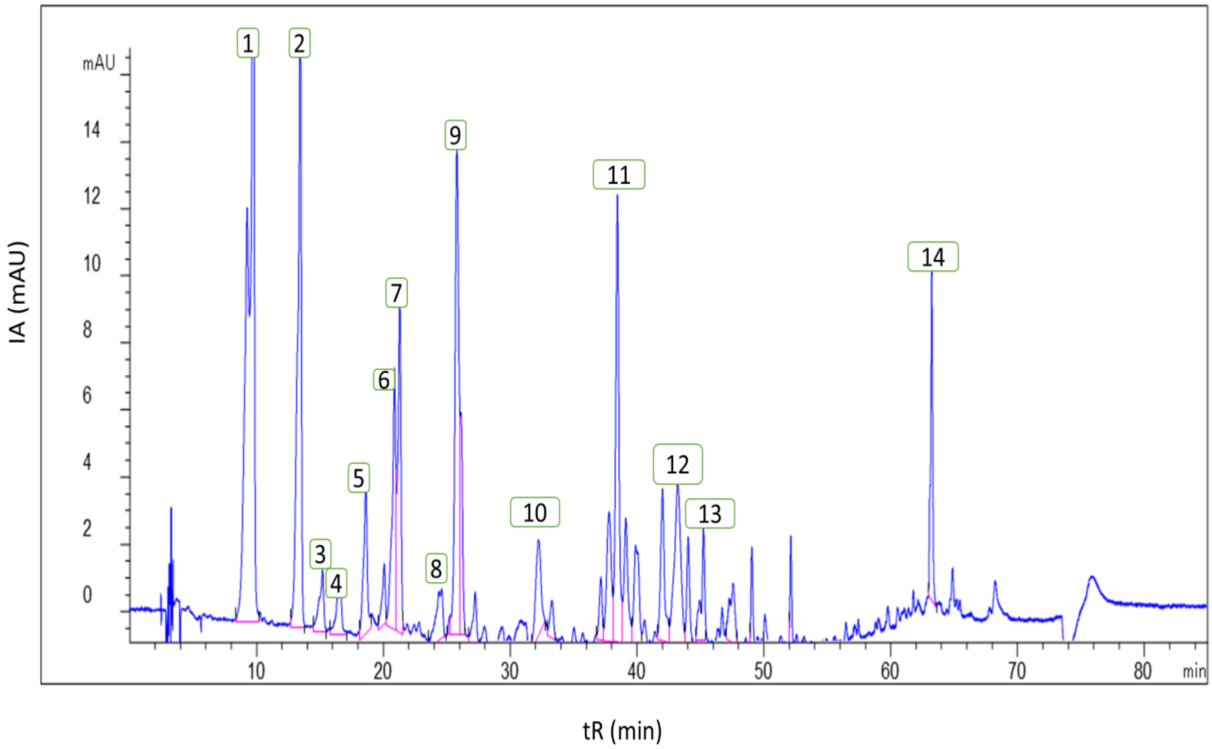

Figure S4. Total ion chromatograms (TICs) from the HPLC analysis of the phenolic compounds recorded at 280 nm in Control 2 (non-deep-fried supplemented EVOO cv. Manzanilla). (1: Hydroxytyrosol; 2: Tyrosol; 3: Chlorogenic acid; 4: Vanillic acid; 5: Homovanillic acid; 6: Caffeic acid; 7: Internal standard (syringic acid); 8: Vanillin; 9: Verbascoside; 10: *p*-Coumaric acid; 11: Decarboxymethyl oleuropein aglycone, dialdehyde form (DOAD); 12: Pinoresinol; 13: 1-Acetoxypinoresinol; and 14: Apigenin).

where: IA: The intensity of absorbance, mAU: milli-Absorbance Units, tR: retention time.

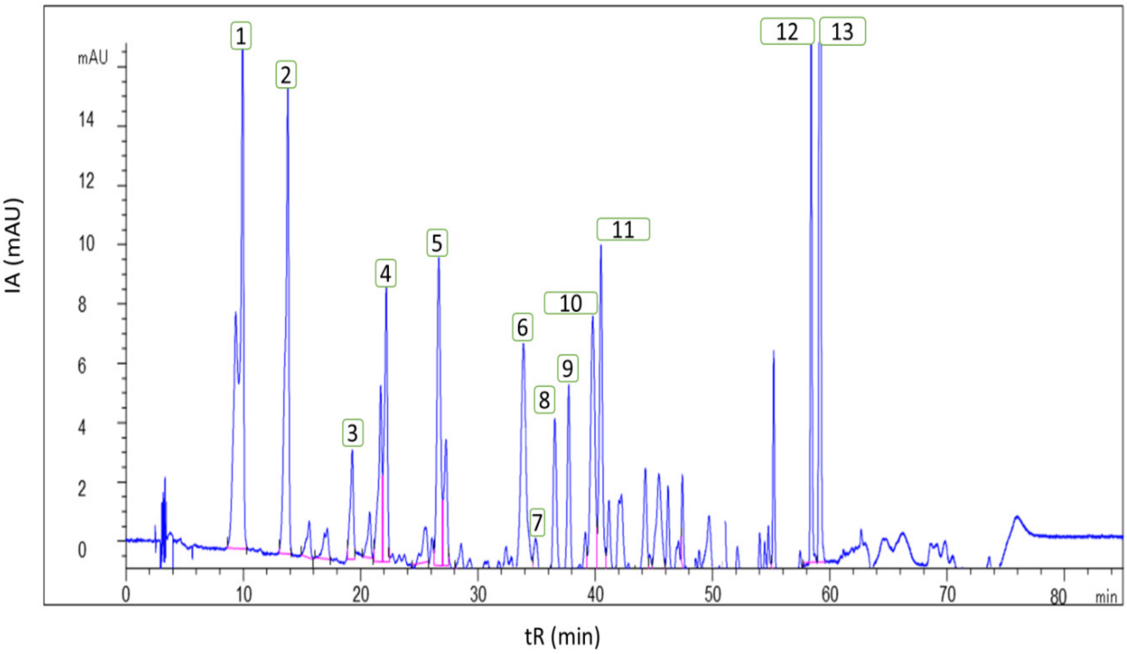

Figure S5. Total ion chromatograms (TICs) from the HPLC analysis of the phenolic compounds recorded at 280 nm in Experiment 1 (Manzanilla oil deep-fried at 170 °C for 3 h with polyphenols supplementation). (1: Hydroxytyrosol; 2: Tyrosol; 3: Homovanillic acid; 4: Internal standard (syringic acid); 5: Verbascoside; 6: Decarboxymethyl oleuropein aglycone, dialdehyde form (DOAD); 7: Decarboxymethyl oleuropein aglycone, oxidized dialdehyde form (oxidized phenolic compound 1) (DOAOD); 8: Oleuropein aglycone, dialdehyde form (OAD); 9: Decarboxymethyl ligstroside aglycone, oxidized dialdehyde form (oxidized phenolic compound 2) (DLAOD); 10: Decarboxymethyl ligstroside aglycone, dialdehyde form (DLAD); 11: Pinoresinol; 12: Oleuropein aglycone, oxidized aldehyde and hydroxylic form (oxidized phenolic compound 3) (OAOAH); and 13: LAOAH: Ligstroside aglycone, oxidized aldehyde and hydroxylic form (oxidized phenolic compound 4) (LAOAH)).

where: IA: The intensity of absorbance, mAU: milli-Absorbance Units, tR: retention time.

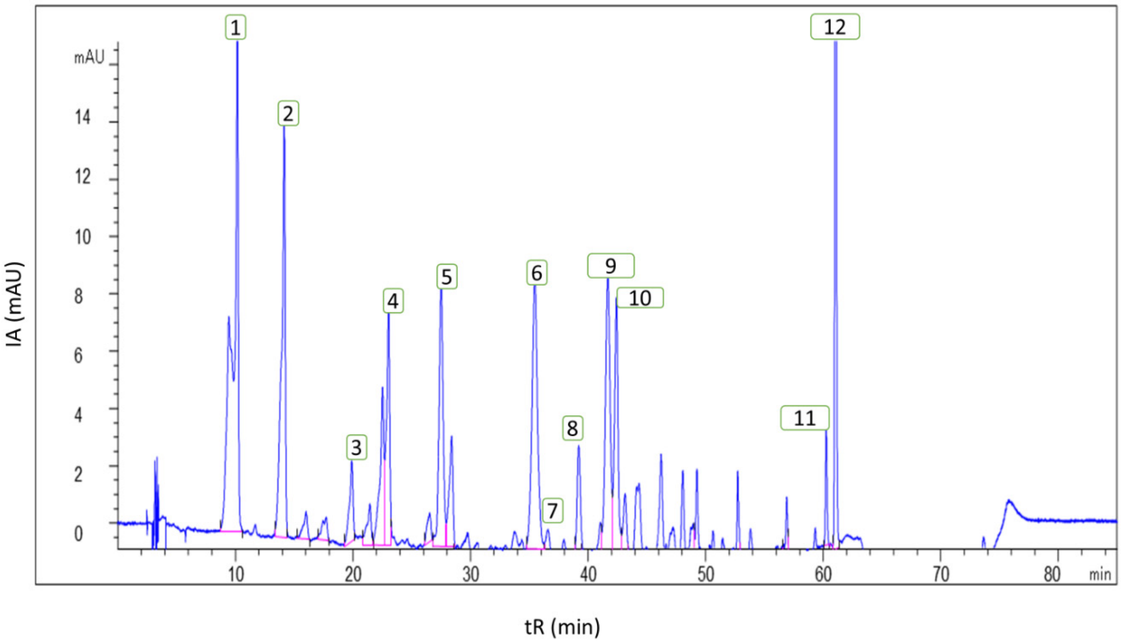

Figure S6. Total ion chromatograms of the phenolic compounds in Experiment 2 (Manzanilla oil deep-fried at 210 °C for 3 h with polyphenols supplementation). (1: Hydroxytyrosol; 2: Tyrosol; 3: Homovanillic acid; 4: Internal standard (syringic acid); 5: Verbascoside; 6: Decarboxymethyl oleuropein aglycone, dialdehyde form (DOAD); 7: Decarboxymethyl oleuropein aglycone, oxidized dialdehyde form (oxidized phenolic compound 1) (DOAOD); 8: Decarboxymethyl ligstroside aglycone, oxidized dialdehyde form (oxidized phenolic compound 2) (DLAOD); 9: Decarboxymethyl ligstroside aglycone, dialdehyde form (DLAD); 10: Pinoresinol; 11: Oleuropein aglycone, oxidized aldehyde and hydroxylic form (oxidized phenolic compound 3) (OAOAH); and 12: LAOAH: Ligstroside aglycone, oxidized aldehyde and hydroxylic form (oxidized phenolic compound 4) (LAOAH)).

where: IA: The intensity of absorbance, mAU: milli-Absorbance Units, tR: retention time.

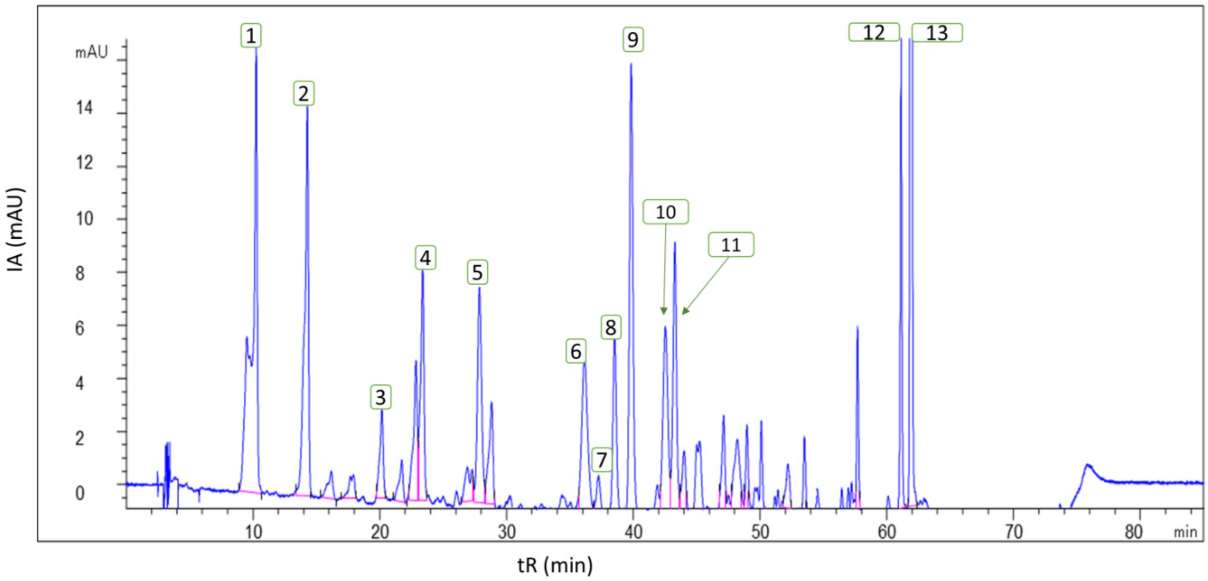

Figure S7. Total ion chromatograms (TICs) from the HPLC analysis of the phenolic compounds recorded at 280 nm in Experiment 3 (Manzanilla oil deep-fried at 170 °C for 6 h with polyphenols supplementation). (1: Hydroxytyrosol; 2: Tyrosol; 3: Homovanillic acid; 4: Internal standard (syringic acid); 5: Verbascoside; 6: Decarboxymethyl oleuropein aglycone, dialdehyde form (DOAD); 7: Decarboxymethyl oleuropein aglycone, oxidized dialdehyde form (oxidized phenolic compound 1) (DOAOD); 8: Oleuropein aglycone, dialdehyde form (OAD); 9: Decarboxymethyl ligstroside aglycone, oxidized dialdehyde form (oxidized phenolic compound 2) (DLAOD); 10: Decarboxymethyl ligstroside aglycone, dialdehyde form (DLAD); 11: Pinoresinol; 12: Oleuropein aglycone, oxidized aldehyde and hydroxylic form (oxidized phenolic compound 3) (OAOAH); and 13: LAOAH: Ligstroside aglycone, oxidized aldehyde and hydroxylic form (oxidized phenolic compound 4) (LAOAH)).

where: IA: The intensity of absorbance, mAU: milli-Absorbance Units, tR: retention time.

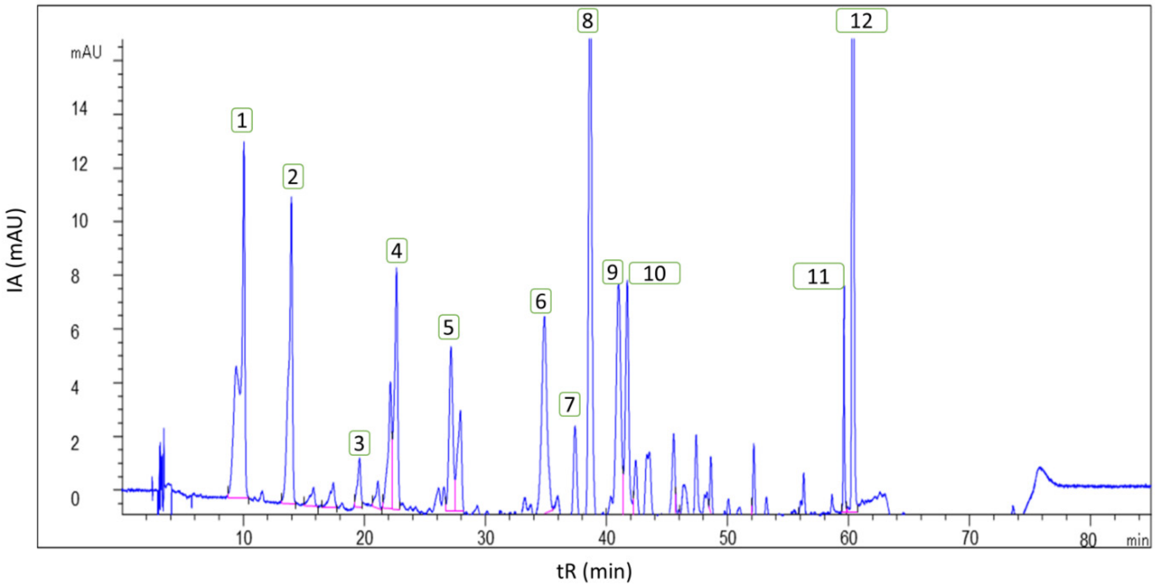

Figure S8. Total ion chromatograms (TICs) from the HPLC analysis of the phenolic compounds recorded at 280 nm in Experiment 4 (Manzanilla oil deep-fried at 210 ℃ for 6 h with polyphenols supplementation). (1: Hydroxytyrosol; 2: Tyrosol; 3: Homovanillic acid; 4: Internal standard (syringic acid); 5: Verbascoside; 6: Decarboxymethyl oleuropein aglycone, dialdehyde form (DOAD); 7: Oleuropein aglycone, dialdehyde form (OAD); 8: Decarboxymethyl ligstroside aglycone, oxidized dialdehyde form (oxidized phenolic compound 2) (DLAOD); 9: Decarboxymethyl ligstroside aglycone, dialdehyde form (DLAD); 10: Pinoresinol; 11: Oleuropein aglycone, oxidized aldehyde and hydroxylic form (oxidized phenolic compound 3) (OAOAH); and 12: LAOAH: Ligstroside aglycone, oxidized aldehyde and hydroxylic form (oxidized phenolic compound 4) (LAOAH)).

where: IA: The intensity of absorbance, mAU: milli-Absorbance Units, tR: retention time.

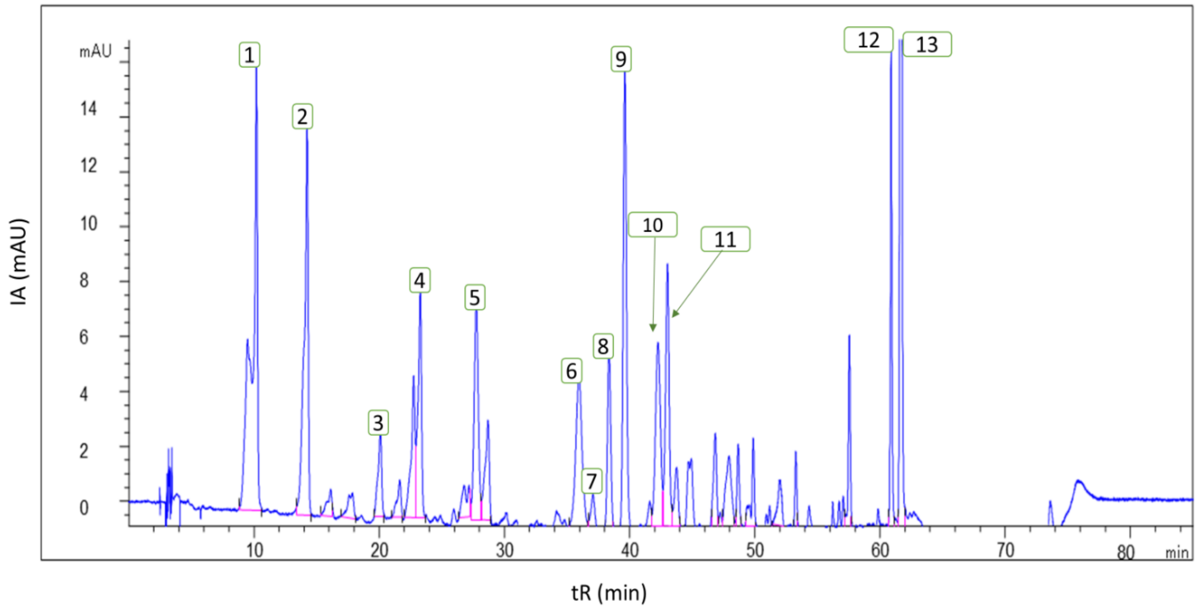

Figure S9. Total ion chromatograms (TICs) from the HPLC analysis of the phenolic compounds recorded at 280 nm in Experiment 5 (Manzanilla oil deep-fried at 170 °C for 12 h with polyphenols supplementation). (1: Hydroxytyrosol; 2: Tyrosol; 3: Homovanillic acid; 4: Internal standard (syringic acid); 5: Verbascoside; 6: Decarboxymethyl oleuropein aglycone, dialdehyde form (DOAD); 7: Decarboxymethyl oleuropein aglycone, oxidized dialdehyde form (oxidized phenolic compound 1) (DOAOD); 8: Oleuropein aglycone, dialdehyde form (OAD); 9: Decarboxymethyl ligstroside aglycone, oxidized dialdehyde form (oxidized phenolic compound 2) (DLAOD); 10: Decarboxymethyl ligstroside aglycone, dialdehyde form (DLAD); 11: Pinoresinol; 12: Oleuropein aglycone, oxidized aldehyde and hydroxylic form (oxidized phenolic compound 3) (OAOAH); and 13: LAOAH: Ligstroside aglycone, oxidized aldehyde and hydroxylic form (oxidized phenolic compound 4) (LAOAH)).

where: IA: The intensity of absorbance, mAU: milli-Absorbance Units, tR: retention time.

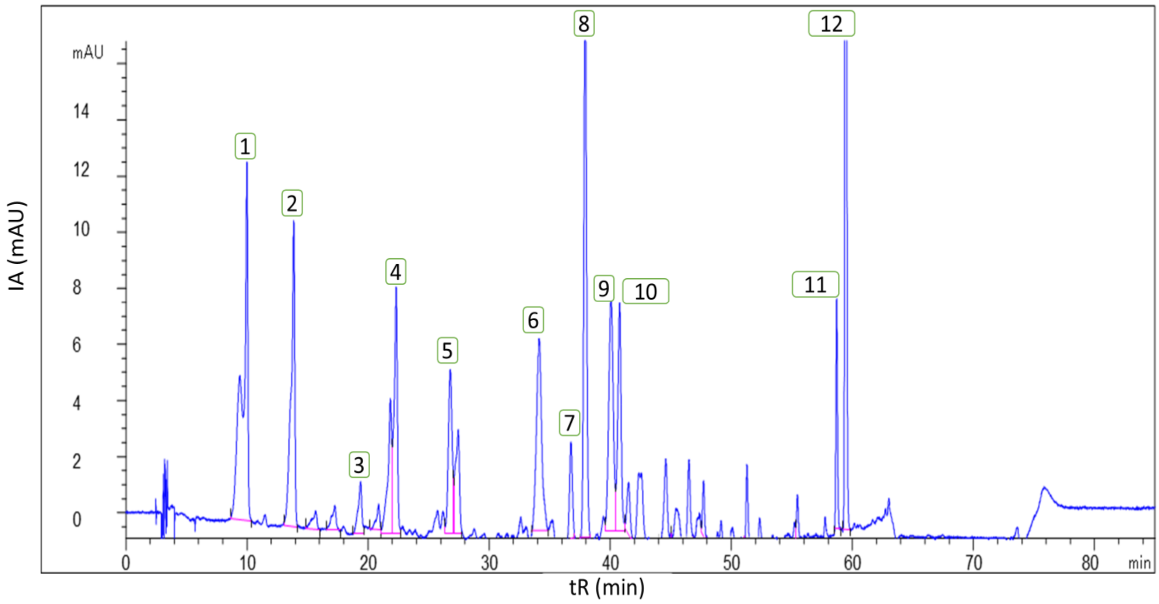

Figure S10. Total ion chromatograms (TICs) from the HPLC analysis of the phenolic compounds recorded at 280 nm in Experiment 6 (Manzanilla oil deep-fried at 210 °C for 12 h with polyphenols supplementation). (1: Hydroxytyrosol; 2: Tyrosol; 3: Homovanillic acid; 4: Internal standard (syringic acid); 5: Verbascoside; 6: Decarboxymethyl oleuropein aglycone, dialdehyde form (DOAD); 7: Oleuropein aglycone, dialdehyde form (OAD); 8: Decarboxymethyl ligstroside aglycone, oxidized dialdehyde form (oxidized phenolic compound 2) (DLAOD); 9: Decarboxymethyl ligstroside aglycone, dialdehyde form (DLAD); 10: Pinoresinol; 11: Oleuropein aglycone, oxidized aldehyde and hydroxylic form (oxidized phenolic compound 3) (OAOAH); and 12: LAOAH: Ligstroside aglycone, oxidized aldehyde and hydroxylic form (oxidized phenolic compound 4) (LAOAH)).

where: IA: The intensity of absorbance, mAU: milli-Absorbance Units, tR: retention time.

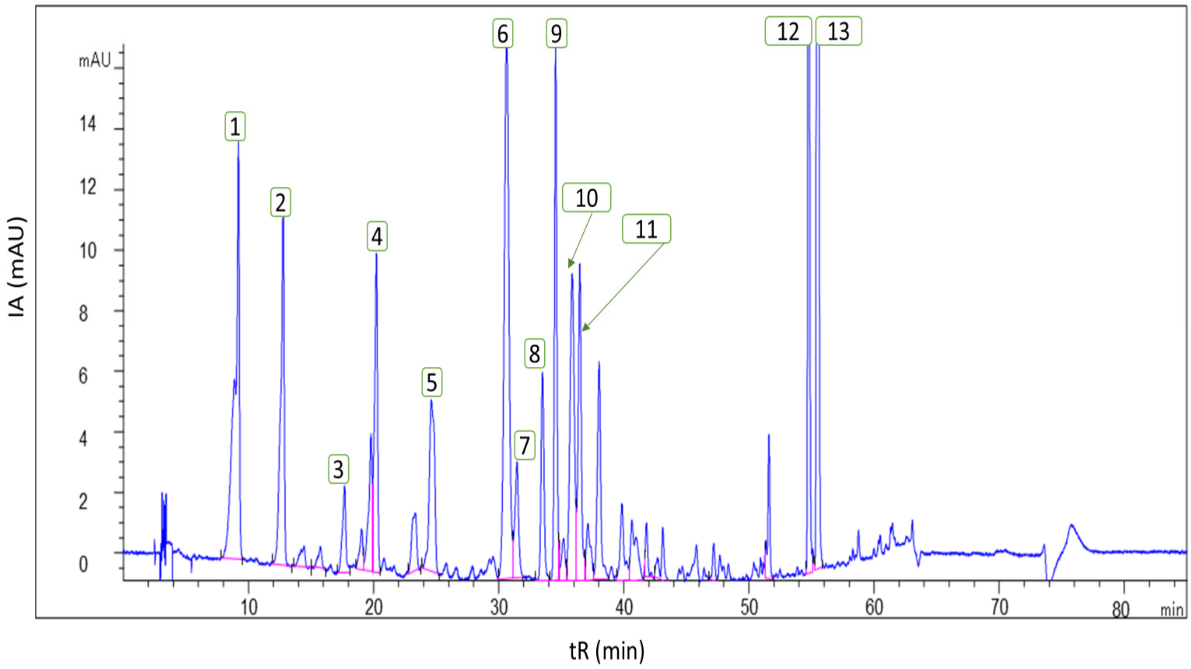

Figure S11. Total ion chromatograms (TICs) from the HPLC analysis of the phenolic compounds recorded at 280 nm in Experiment 7 (Manzanilla oil deep-fried at 170 °C for 18 h with polyphenols supplementation). (1: Hydroxytyrosol; 2: Tyrosol; 3: Homovanillic acid; 4: Internal standard (syringic acid); 5: Verbascoside; 6: Decarboxymethyl oleuropein aglycone, dialdehyde form (DOAD); 7: Decarboxymethyl oleuropein aglycone, oxidized dialdehyde form (oxidized phenolic compound 1) (DOAOD); 8: Oleuropein aglycone, dialdehyde form (OAD); 9: Decarboxymethyl ligstroside aglycone, oxidized dialdehyde form (oxidized phenolic compound 2) (DLAOD); 10: Decarboxymethyl ligstroside aglycone, dialdehyde form (DLAD); 11: Pinoresinol; 12: Oleuropein aglycone, oxidized aldehyde and hydroxylic form (oxidized phenolic compound 3) (OAOAH); and 13: Ligstroside aglycone, oxidized aldehyde and hydroxylic form (oxidized phenolic compound 4) (LAOAH)).

where: IA: The intensity of absorbance, mAU: milli-Absorbance Units, tR: retention time.

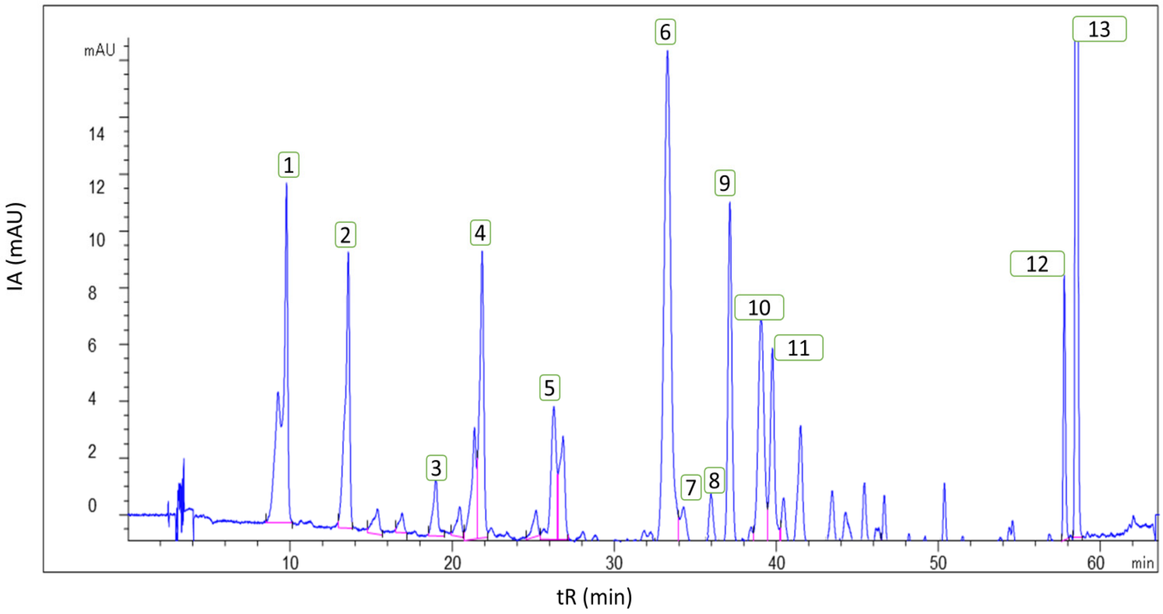

Figure S12. Total ion chromatograms (TICs) from the HPLC analysis of the phenolic compounds recorded at 280 nm in Experiment 8 (Manzanilla oil deep-fried at 210 °C for 18 h with polyphenols supplementation). (1: Hydroxytyrosol; 2: Tyrosol; 3: Homovanillic acid; 4: Internal standard (syringic acid); 5: Verbascoside; 6: Decarboxymethyl oleuropein aglycone, dialdehyde form (DOAD); 7: Decarboxymethyl oleuropein aglycone, oxidized dialdehyde form (oxidized phenolic compound 1) (DOAOD); 8: Oleuropein aglycone, dialdehyde form (OAD); 9: Decarboxymethyl ligstroside aglycone, oxidized dialdehyde form (oxidized phenolic compound 2) (DLAOD); 10: Decarboxymethyl ligstroside aglycone, dialdehyde form (DLAD); 11: Pinoresinol; 12: Oleuropein aglycone, oxidized aldehyde and hydroxylic form (oxidized phenolic compound 3) (OAOAH); and 13: Ligstroside aglycone, oxidized aldehyde and hydroxylic form (oxidized phenolic compound 4) (LAOAH)).

where: IA: The intensity of absorbance, mAU: milli-Absorbance Units, tR: retention time.

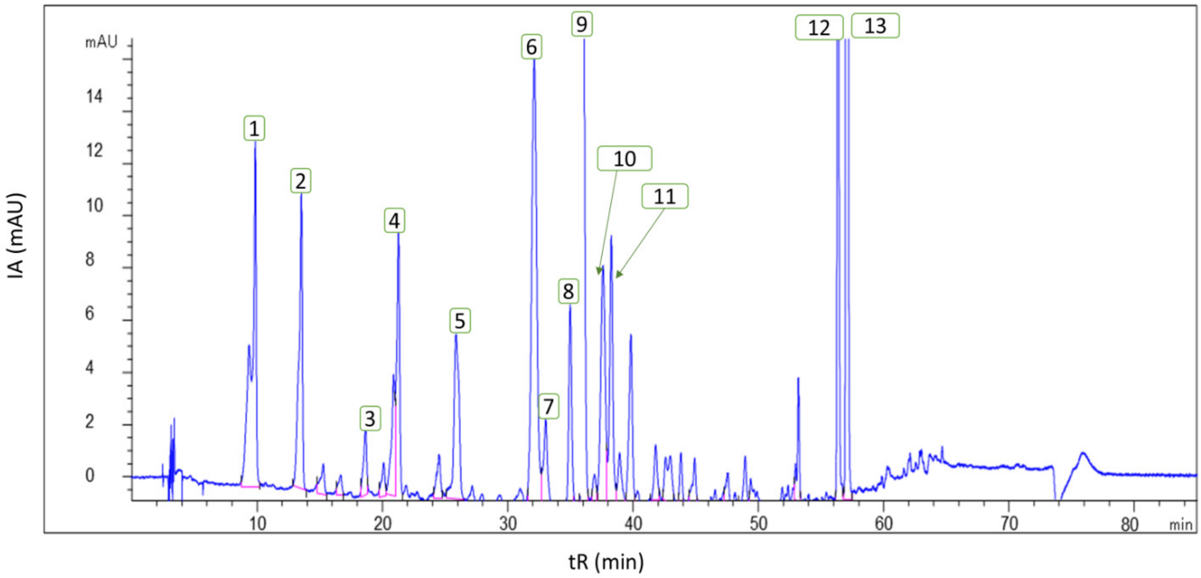

Figure S13. Total ion chromatograms (TICs) from the HPLC analysis of the phenolic compounds recorded at 280 nm in Experiment 9 (Manzanilla oil deep-fried at 170 °C for 24 h with polyphenols supplementation). (1: Hydroxytyrosol; 2: Tyrosol; 3: Homovanillic acid; 4: Internal standard (syringic acid); 5: Verbascoside; 6: Decarboxymethyl oleuropein aglycone, dialdehyde form (DOAD); 7: Decarboxymethyl oleuropein aglycone, oxidized dialdehyde form (oxidized phenolic compound 1) (DOAOD); 8: Oleuropein aglycone, dialdehyde form (OAD); 9: Decarboxymethyl ligstroside aglycone, oxidized dialdehyde form (oxidized phenolic compound 2) (DLAOD); 10: Decarboxymethyl ligstroside aglycone, dialdehyde form (DLAD); 11: Pinoresinol; 12: Oleuropein aglycone, oxidized aldehyde and hydroxylic form (oxidized phenolic compound 3) (OAOAH); and 13: Ligstroside aglycone, oxidized aldehyde and hydroxylic form (oxidized phenolic compound 4) (LAOAH)).

where: IA: The intensity of absorbance, mAU: milli-Absorbance Units, tR: retention time.

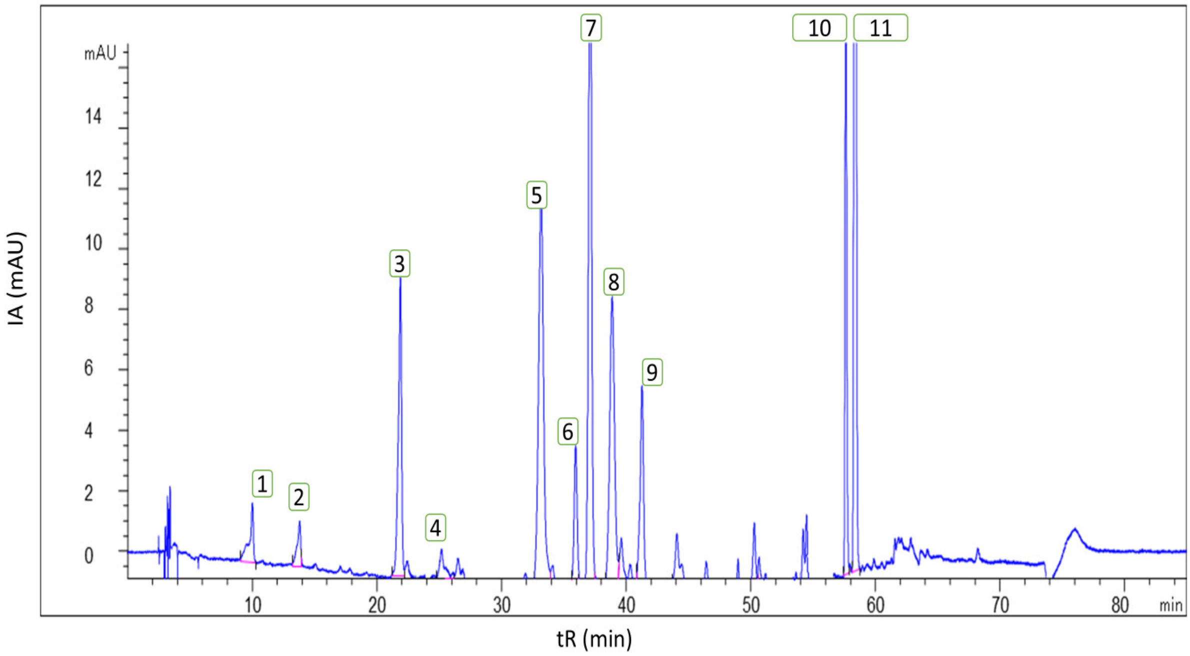

Figure S14. Total ion chromatograms (TICs) from the HPLC analysis of the phenolic compounds recorded at 280 nm in Experiment 10 (Manzanilla oil deep-fried at 210 °C for 24 h with polyphenols supplementation). (1: Hydroxytyrosol; 2: Tyrosol; 3: Internal standard (syringic acid); 4: Verbascoside; 5: Decarboxymethyl oleuropein aglycone, dialdehyde form (DOAD); 7: Decarboxymethyl oleuropein aglycone, oxidized dialdehyde form (oxidized phenolic compound 1) (DOAOD); 6: Oleuropein aglycone, dialdehyde form (OAD); 7: Decarboxymethyl ligstroside aglycone, oxidized dialdehyde form (oxidized phenolic compound 2) (DLAOD); 8: Decarboxymethyl ligstroside aglycone, dialdehyde form (DLAD); 9: Pinoresinol; 10: Oleuropein aglycone, oxidized aldehyde and hydroxylic form (oxidized phenolic compound 3) (OAOAH); and 11: Ligstroside aglycone, oxidized aldehyde and hydroxylic form (oxidized phenolic compound 4) (LAOAH)).

where: IA: The intensity of absorbance, mAU: milli-Absorbance Units, tR: retention time.

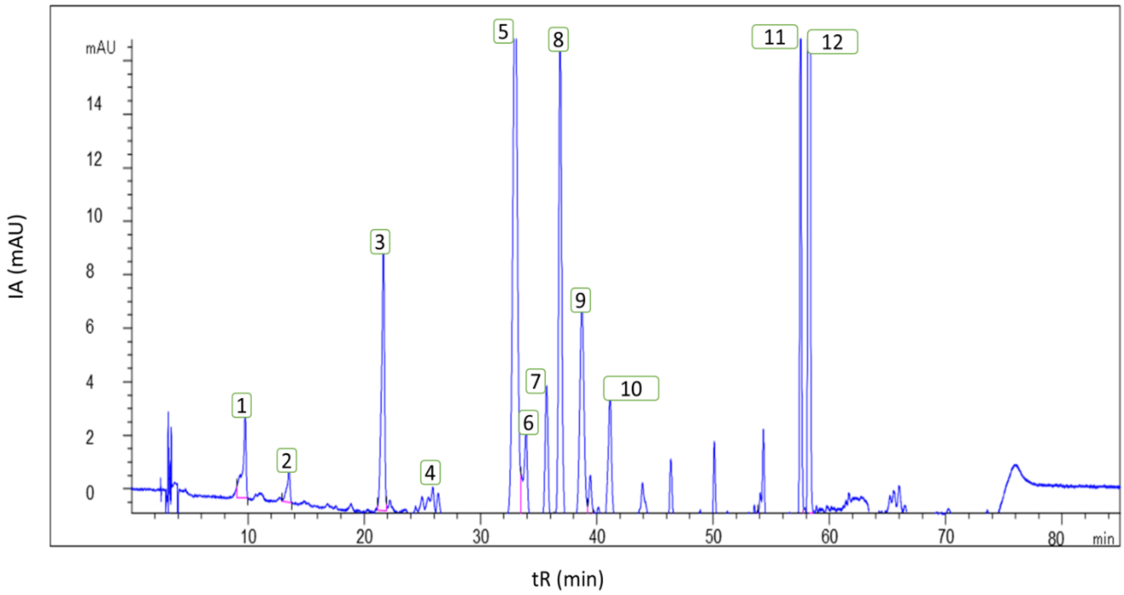

Figure S15. Total ion chromatograms (TICs) from the HPLC analysis of the phenolic compounds recorded at 280 nm in Experiment 11 (Manzanilla oil deep-fried at 170 °C for 48 h with polyphenols supplementation). (1: Hydroxytyrosol; 2: Tyrosol; 3: Internal standard (syringic acid); 4: Verbascoside; 5: Decarboxymethyl oleuropein aglycone, dialdehyde form (DOAD); 6: Decarboxymethyl oleuropein aglycone, oxidized dialdehyde form (oxidized phenolic compound 1) (DOAOD); 7: Oleuropein aglycone, dialdehyde form (OAD); 8: Decarboxymethyl ligstroside aglycone, oxidized dialdehyde form (oxidized phenolic compound 2) (DLAOD); 9: Decarboxymethyl ligstroside aglycone, dialdehyde form (DLAD); 10: Pinoresinol; 11: Oleuropein aglycone, oxidized aldehyde and hydroxylic form (oxidized phenolic compound 3) (OAOAH); and 12: Ligstroside aglycone, oxidized aldehyde and hydroxylic form (oxidized phenolic compound 4) (LAOAH)).

where: IA: The intensity of absorbance, mAU: milli-Absorbance Units, tR: retention time.

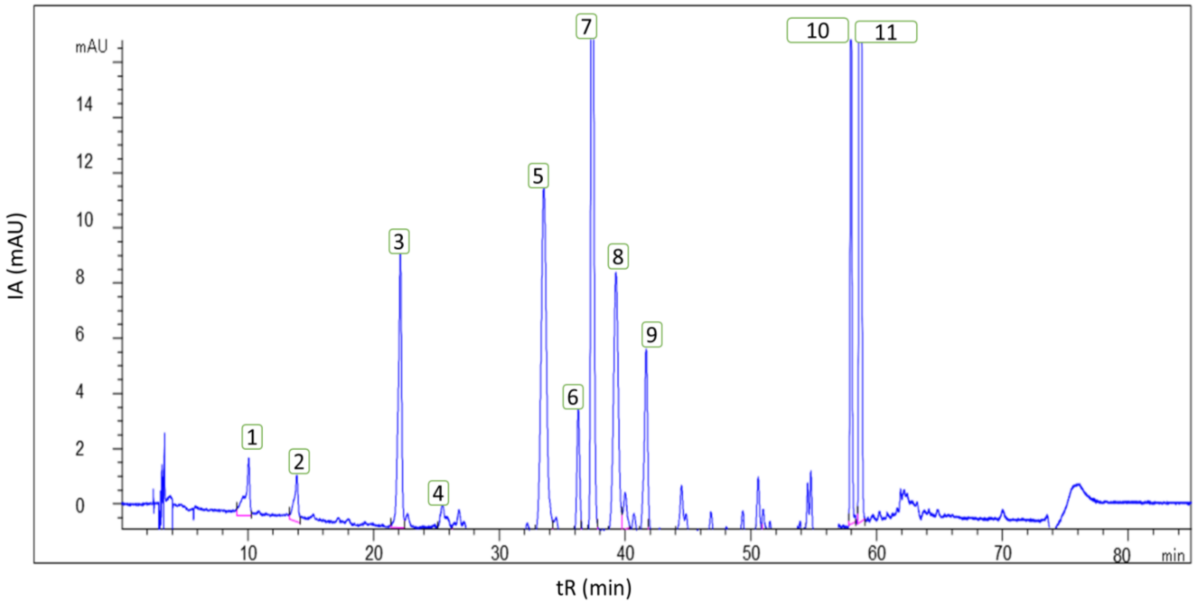

Figure S16. Total ion chromatograms (TICs) from the HPLC analysis of the phenolic compounds recorded at 280 nm in Experiment 12 (Manzanilla oil deep-fried at 210 °C for 48 h with polyphenols supplementation). (1: Hydroxytyrosol; 2: Tyrosol; 3: Internal standard (syringic acid); 4: Verbascoside; 5: Decarboxymethyl oleuropein aglycone, dialdehyde form (DOAD); 6: Oleuropein aglycone, dialdehyde form (OAD); 7: Decarboxymethyl ligstroside aglycone, oxidized dialdehyde form (oxidized phenolic compound 2) (DLAOD); 8: Decarboxymethyl ligstroside aglycone, dialdehyde form (DLAD); 9: Pinoresinol; 10: Oleuropein aglycone, oxidized aldehyde and hydroxylic form (oxidized phenolic compound 3) (OAOAH); and 11: Ligstroside aglycone, oxidized aldehyde and hydroxylic form (oxidized phenolic compound 4) (LAOAH)).

where: IA: The intensity of absorbance, mAU: milli-Absorbance Units, tR: retention time.

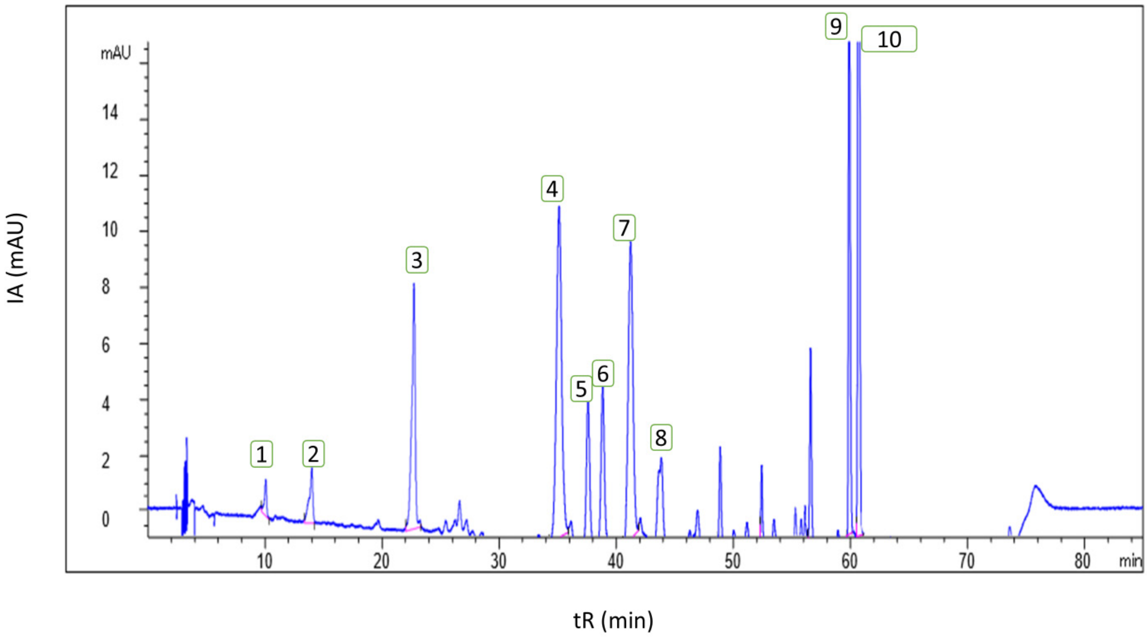

Figure S17. Total ion chromatograms (TICs) from the HPLC analysis of the phenolic compounds recorded at 280 nm in Experiment 13 (Manzanilla oil deep-fried at 170 °C for 3 h without polyphenols supplementation). (1: Hydroxytyrosol; 2: Tyrosol; 3: Internal standard (syringic acid); 4: Decarboxymethyl oleuropein aglycone, dialdehyde form (DOAD); 5: Oleuropein aglycone, dialdehyde form (OAD); 6: Decarboxymethyl ligstroside aglycone, oxidized dialdehyde form (oxidized phenolic compound 2) (DLAOD); 7: Decarboxymethyl ligstroside aglycone, dialdehyde form (DLAD); 8: Acetoxypinoresinol; 9: Oleuropein aglycone, oxidized aldehyde and hydroxylic form (oxidized phenolic compound 3) (OAOAH); and 10: Ligstroside aglycone, oxidized aldehyde and hydroxylic form (oxidized phenolic compound 4) (LAOAH)).

where: IA: The intensity of absorbance, mAU: milli-Absorbance Units, tR: Retention time.

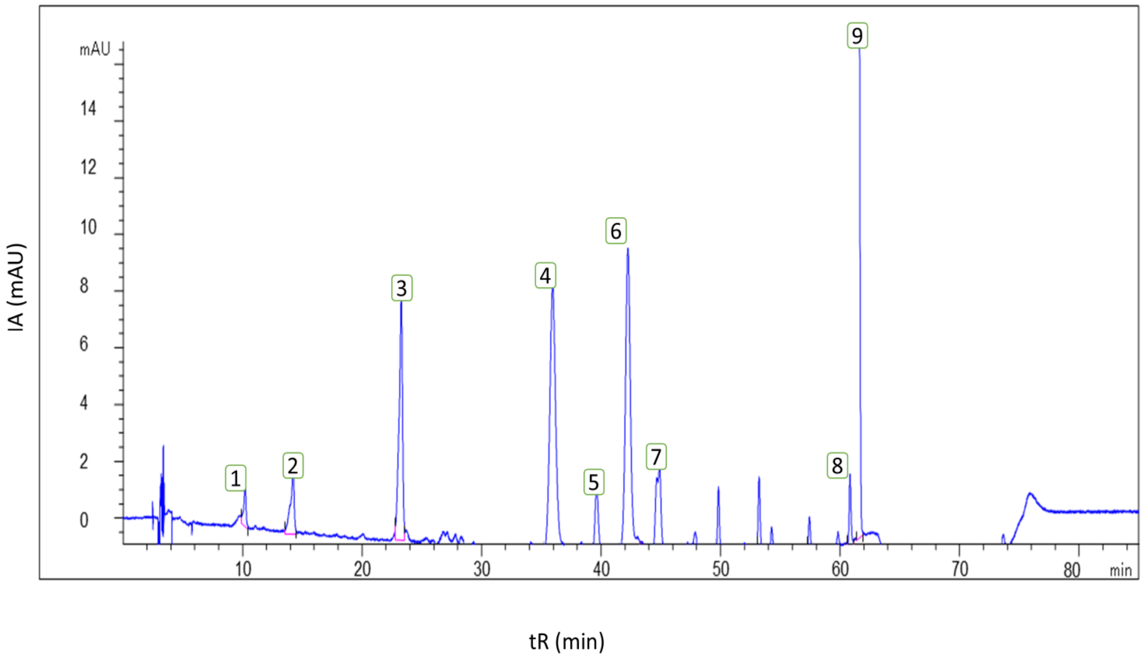

Figure S18. Total ion chromatograms (TICs) from the HPLC analysis of the phenolic compounds recorded at 280 nm in Experiment 14 (Manzanilla oil deep-fried at 210 °C for 3 h without polyphenols supplementation). (1: Hydroxytyrosol; 2: Tyrosol; 3: Internal standard (syringic acid); 4: Decarboxymethyl oleuropein aglycone, dialdehyde form (DOAD); 5: Decarboxymethyl ligstroside aglycone, oxidized dialdehyde form (oxidized phenolic compound 2) (DLAOD); 6: Decarboxymethyl ligstroside aglycone, dialdehyde form (DLAD); 7: Acetoxypinoresinol; 8: Oleuropein aglycone, oxidized aldehyde and hydroxylic form (oxidized phenolic compound 3) (OAOAH); and 9: Ligstroside aglycone, oxidized aldehyde and hydroxylic form (oxidized phenolic compound 4) (LAOAH)).

where: IA: The intensity of absorbance, mAU: milli-Absorbance Units, tR: Retention time.

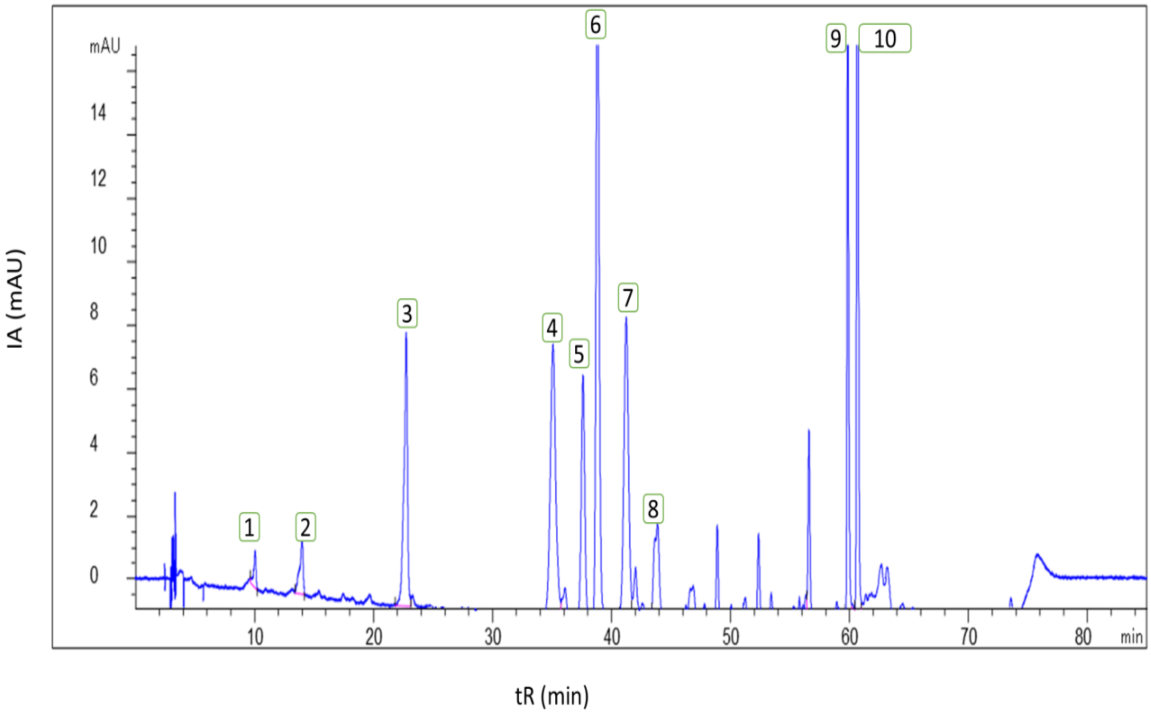

Figure S19. Total ion chromatograms (TICs) from the HPLC analysis of the phenolic compounds recorded at 280 nm in Experiment 15 (Manzanilla oil deep-fried at 170 °C for 6 h without polyphenols supplementation). (1: Hydroxytyrosol; 2: Tyrosol; 3: Internal standard (syringic acid); 4: Decarboxymethyl oleuropein aglycone, dialdehyde form (DOAD); 5: Oleuropein aglycone, dialdehyde form (OAD); 6: Decarboxymethyl ligstroside aglycone, oxidized dialdehyde form (oxidized phenolic compound 2) (DLAOD); 7: Decarboxymethyl ligstroside aglycone, dialdehyde form (DLAD); 8: Acetoxypinoresinol; 9: Oleuropein aglycone, oxidized aldehyde and hydroxylic form (oxidized phenolic compound 3) (OAOAH); and 10: Ligstroside aglycone, oxidized aldehyde and hydroxylic form (oxidized phenolic compound 4) (LAOAH)).

where: IA: The intensity of absorbance, mAU: milli-Absorbance Units, tR: Retention time.

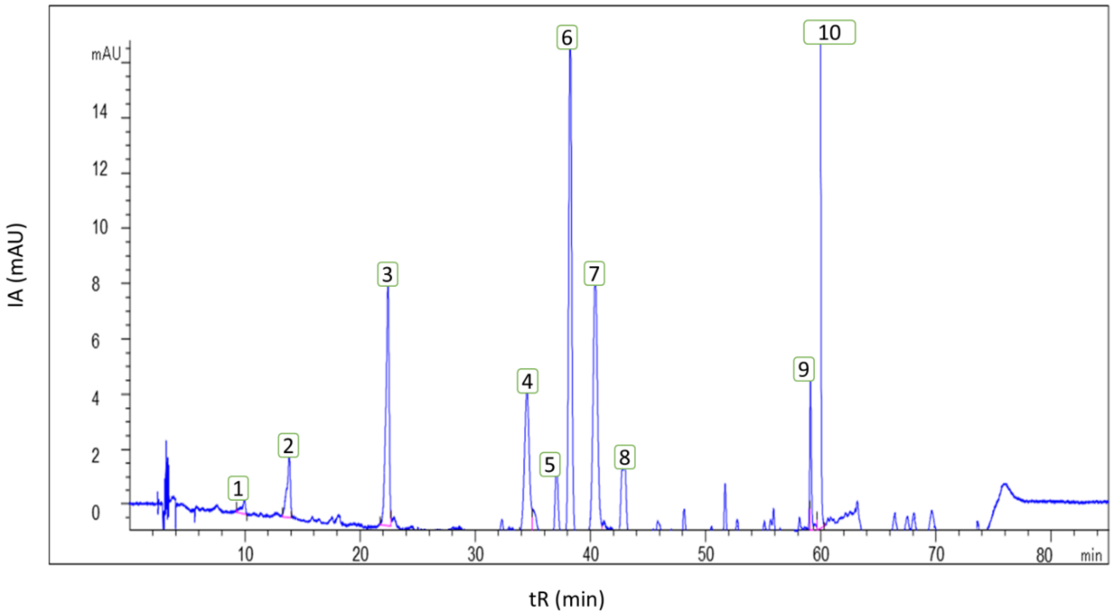

Figure S20. Total ion chromatograms (TICs) from the HPLC analysis of the phenolic compounds recorded at 280 nm in Experiment 16 (Manzanilla oil deep-fried at 210 °C for 6 h without polyphenols supplementation). (1: Hydroxytyrosol; 2: Tyrosol; 3: Internal standard (syringic acid); 4: Decarboxymethyl oleuropein aglycone, dialdehyde form (DOAD); 5: Oleuropein aglycone, dialdehyde form (OAD); 6: Decarboxymethyl ligstroside aglycone, oxidized dialdehyde form (oxidized phenolic compound 2) (DLAOD); 7: Decarboxymethyl ligstroside aglycone, dialdehyde form (DLAD); 8: Acetoxypinoresinol; 9: Oleuropein aglycone, oxidized aldehyde and hydroxylic form (oxidized phenolic compound 3) (OAOAH); and 10: Ligstroside aglycone, oxidized aldehyde and hydroxylic form (oxidized phenolic compound 4) (LAOAH)).

where: IA: The intensity of absorbance, mAU: milli-Absorbance Units, tR: Retention time.

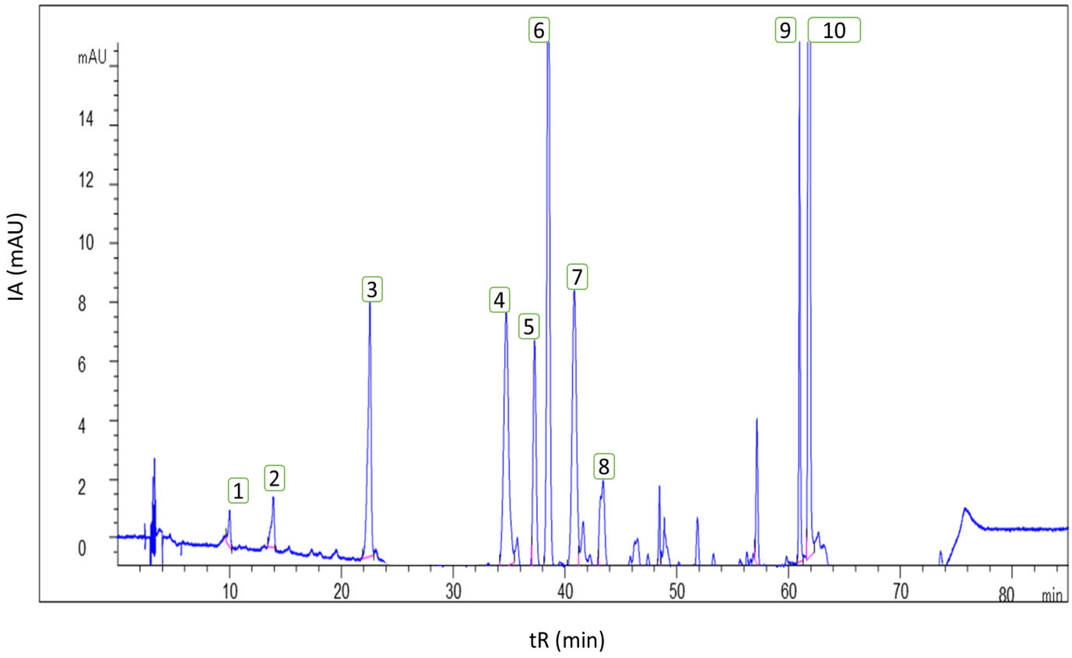

Figure S21. Total ion chromatograms (TICs) from the HPLC analysis of the phenolic compounds recorded at 280 nm in Experiment 17 (Manzanilla oil deep-fried at 170 °C for 12 h without polyphenols supplementation). (1: Hydroxytyrosol; 2: Tyrosol; 3: Internal standard (syringic acid); 4: Decarboxymethyl oleuropein aglycone, dialdehyde form (DOAD); 5: Oleuropein aglycone, dialdehyde form (OAD); 6: Decarboxymethyl ligstroside aglycone, oxidized dialdehyde form (oxidized phenolic compound 2) (DLAOD); 7: Decarboxymethyl ligstroside aglycone, dialdehyde form (DLAD); 8: Acetoxypinoresinol; 9: Oleuropein aglycone, oxidized aldehyde and hydroxylic form (oxidized phenolic compound 3) (OAOAH); and 10: Ligstroside aglycone, oxidized aldehyde and hydroxylic form (oxidized phenolic compound 4) (LAOAH)).

where: IA: The intensity of absorbance, mAU: milli-Absorbance Units, tR: Retention time.

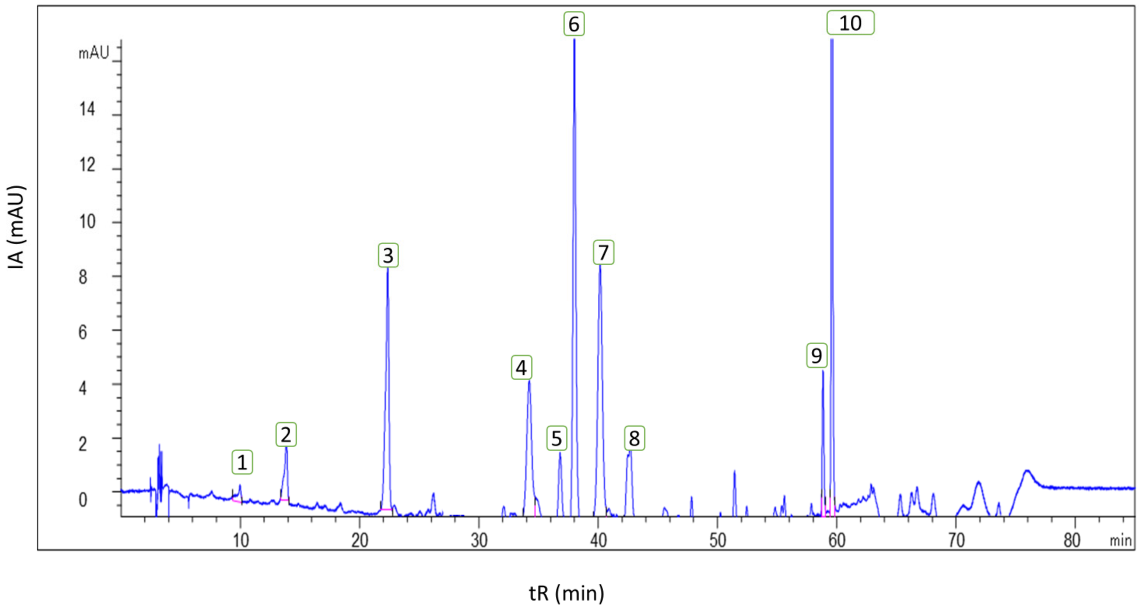

Figure S22. Total ion chromatograms (TICs) from the HPLC analysis of the phenolic compounds recorded at 280 nm in Experiment 18 (Manzanilla oil deep-fried at 210 °C for 12 h without polyphenols supplementation). (1: Hydroxytyrosol; 2: Tyrosol; 3: Internal standard (syringic acid); 4: Decarboxymethyl oleuropein aglycone, dialdehyde form (DOAD); 5: Ligstroside aglycone, dialdehyde form (LAD); 6: Decarboxymethyl ligstroside aglycone, oxidized dialdehyde form (oxidized phenolic compound 2) (DLAOD); 7: Decarboxymethyl ligstroside aglycone, dialdehyde form (DLAD); 8: Acetoxypinoresinol; 9: Oleuropein aglycone, oxidized aldehyde and hydroxylic form (oxidized phenolic compound 3) (OAOAH); and 10: Ligstroside aglycone, oxidized aldehyde and hydroxylic form (oxidized phenolic compound 4) (LAOAH)).

where: IA: The intensity of absorbance, mAU: milli-Absorbance Units, tR: Retention time.

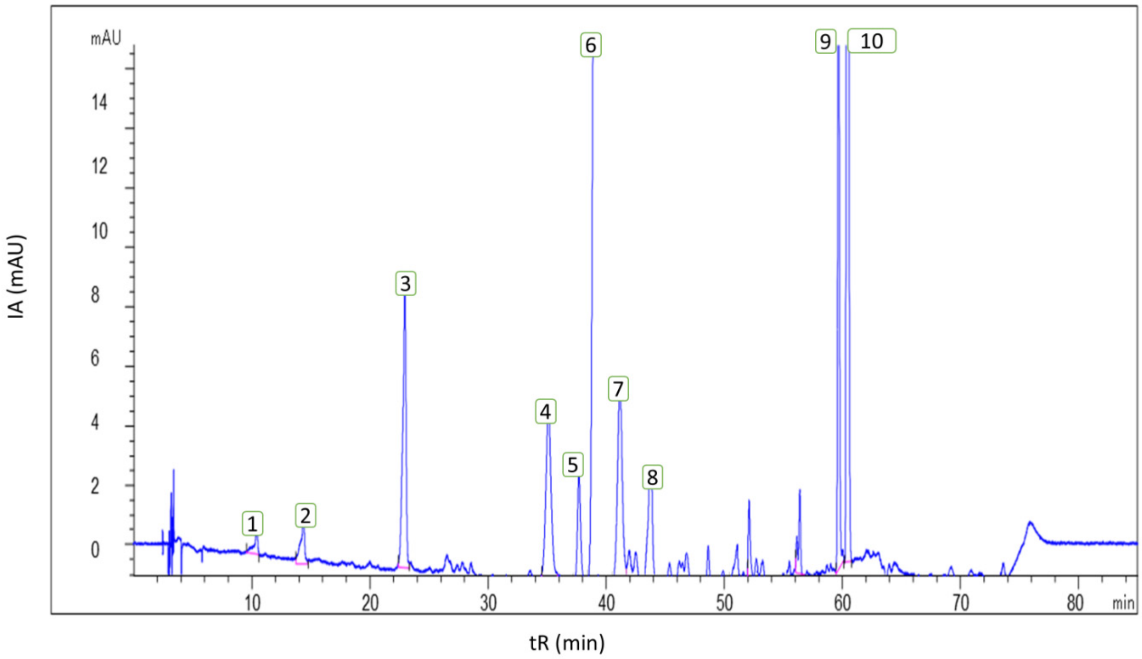

Figure S23. Total ion chromatograms (TICs) from the HPLC analysis of the phenolic compounds recorded at 280 nm in Experiment 19 (Manzanilla oil deep-fried at 170 °C for 18 h without polyphenols supplementation). (1: Hydroxytyrosol; 2: Tyrosol; 3: Internal standard (syringic acid); 4: Decarboxymethyl oleuropein aglycone, dialdehyde form (DOAD); 5: Oleuropein aglycone, dialdehyde form (OAD); 6: Decarboxymethyl ligstroside aglycone, oxidized dialdehyde form (oxidized phenolic compound 2) (DLAOD); 7: Decarboxymethyl ligstroside aglycone, dialdehyde form (DLAD); 8: Acetoxypinoresinol; 9: Oleuropein aglycone, oxidized aldehyde and hydroxylic form (oxidized phenolic compound 3) (OAOAH); and 10: Ligstroside aglycone, oxidized aldehyde and hydroxylic form (oxidized phenolic compound 4) (LAOAH)).

where: IA: The intensity of absorbance, mAU: milli-Absorbance Units, tR: Retention time.

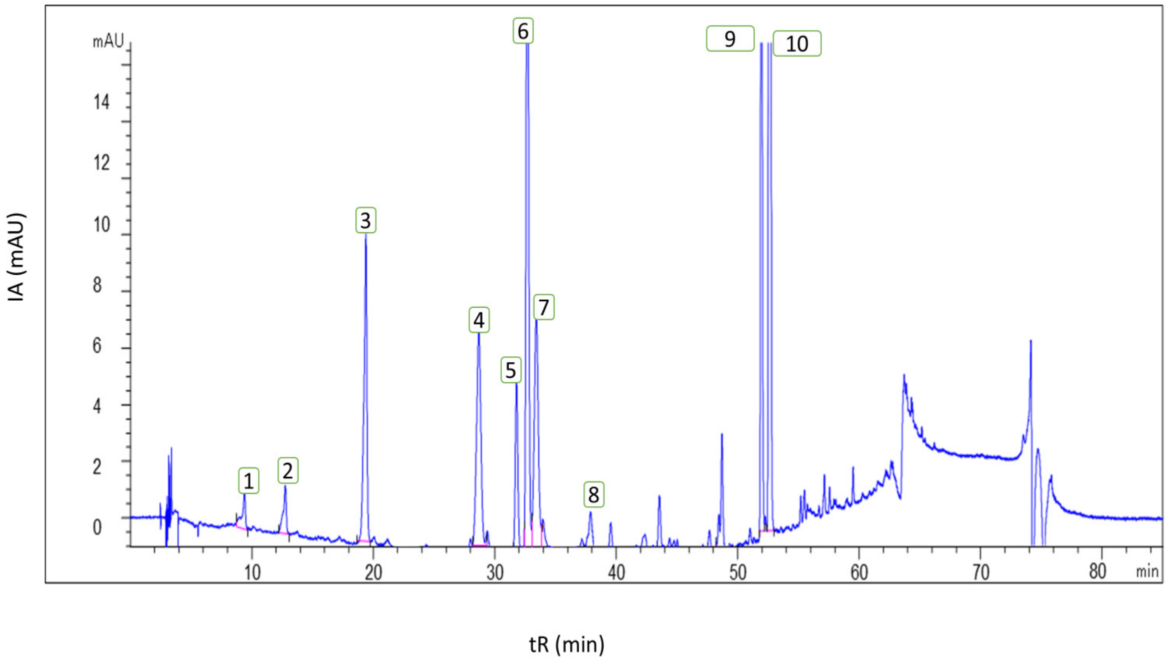

Figure S24. Total ion chromatograms (TICs) from the HPLC analysis of the phenolic compounds recorded at 280 nm in Experiment 20 (Manzanilla oil deep-fried at 210 °C for 18 h without polyphenols supplementation). (1: Hydroxytyrosol; 2: Tyrosol; 3: Internal standard (syringic acid); 4: Decarboxymethyl oleuropein aglycone, dialdehyde form (DOAD); 5: Ligstroside aglycone, dialdehyde form (LAD); 6: Decarboxymethyl ligstroside aglycone, oxidized dialdehyde form (oxidized phenolic compound 2) (DLAOD); 7: Decarboxymethyl ligstroside aglycone, dialdehyde form (DLAD); 8: Acetoxypinoresinol; 9: Oleuropein aglycone, oxidized aldehyde and hydroxylic form (oxidized phenolic compound 3) (OAOAH); and 10: Ligstroside aglycone, oxidized aldehyde and hydroxylic form (oxidized phenolic compound 4) (LAOAH)).

where: IA: The intensity of absorbance, mAU: milli-Absorbance Units, tR: Retention time.

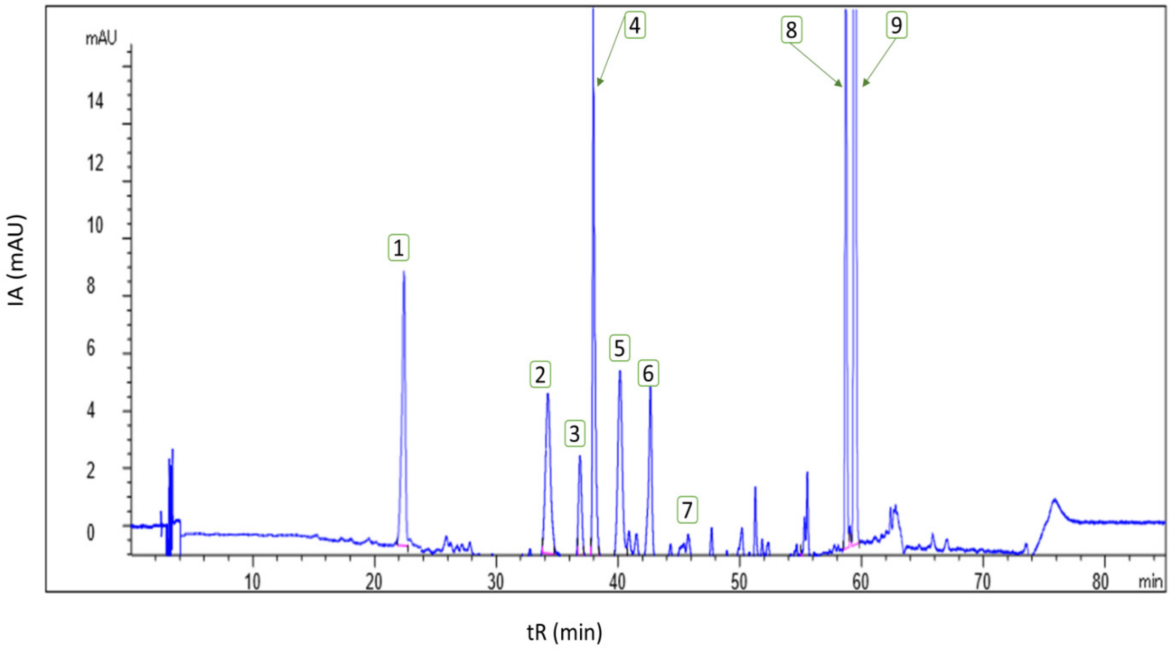

Figure S25. Total ion chromatograms (TICs) from the HPLC analysis of the phenolic compounds recorded at 280 nm in Experiment 21 (Manzanilla oil deep-fried at 170 °C for 24 h without polyphenols supplementation). 1: Internal standard (syringic acid); 2: Decarboxymethyl oleuropein aglycone, dialdehyde form (DOAD); 3: Oleuropein aglycone, dialdehyde form (OAD); 4: Decarboxymethyl ligstroside aglycone, oxidized dialdehyde form (oxidized phenolic compound 2) (DLAOD); 5: Ligstroside aglycone, dialdehyde form (LAD); 6: Decarboxymethyl ligstroside aglycone, dialdehyde form (DLAD); 7: Acetoxypinoresinol; 8: Oleuropein aglycone, oxidized aldehyde and hydroxylic form (oxidized phenolic compound 3) (OAOAH); and 9: Ligstroside aglycone, oxidized aldehyde and hydroxylic form (oxidized phenolic compound 4) (LAOAH)).

where: IA: The intensity of absorbance, mAU: milli-Absorbance Units, tR: Retention time.

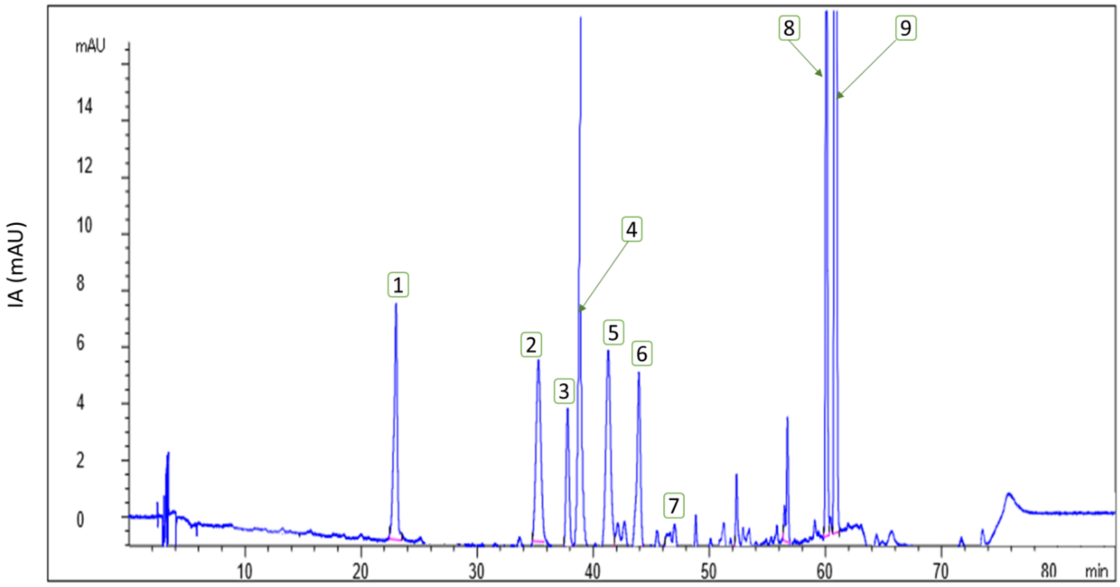

Figure S26. Total ion chromatograms (TICs) from the HPLC analysis of the phenolic compounds recorded at 280 nm in Experiment 22 (Manzanilla oil deep-fried at 210 °C for 24 h without polyphenols supplementation). 1: Internal standard (syringic acid); 2: Decarboxymethyl oleuropein aglycone, dialdehyde form (DOAD); 3: Oleuropein aglycone, dialdehyde form (OAD); 4: Decarboxymethyl ligstroside aglycone, oxidized dialdehyde form (oxidized phenolic compound 2) (DLAOD); 5: Ligstroside aglycone, dialdehyde form (LAD); 6: Decarboxymethyl ligstroside aglycone, dialdehyde form (DLAD); 7: Acetoxypinoresinol; 8: Oleuropein aglycone, oxidized aldehyde and hydroxylic form (oxidized phenolic compound 3) (OAOAH); and 9: Ligstroside aglycone, oxidized aldehyde and hydroxylic form (oxidized phenolic compound 4) (LAOAH)).

where: IA: The intensity of absorbance, mAU: milli-Absorbance Units, tR: Retention time.

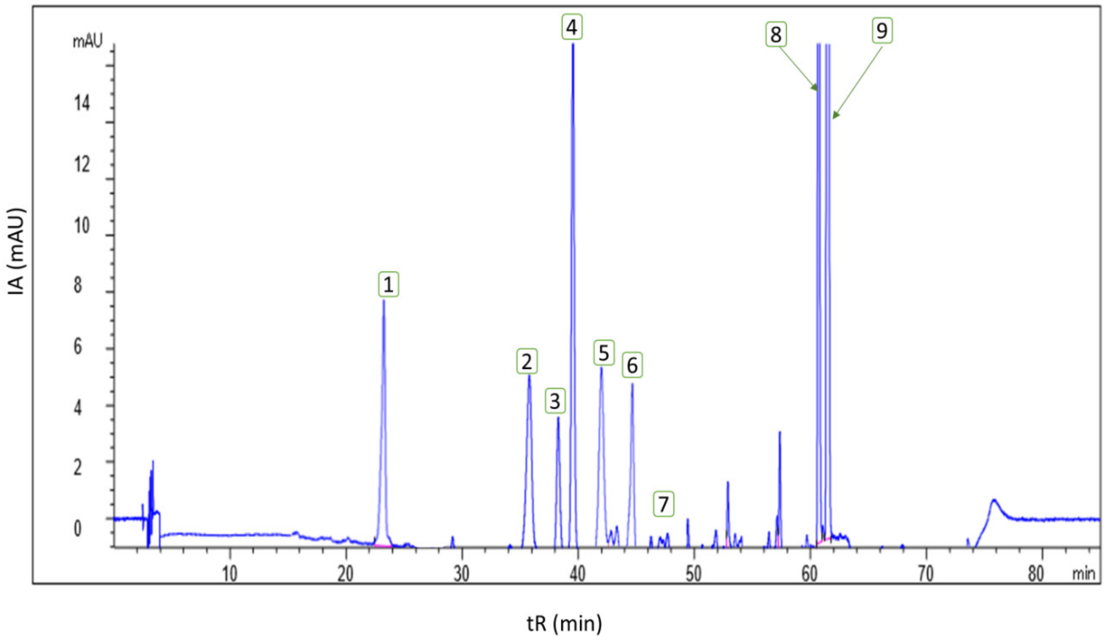

Figure S27. Total ion chromatograms (TICs) from the HPLC analysis of the phenolic compounds recorded at 280 nm in Experiment 23 (Manzanilla oil deep-fried at 170 °C for 48 h without polyphenols supplementation). 1: Internal standard (syringic acid); 2: Decarboxymethyl oleuropein aglycone, dialdehyde form (DOAD); 3: Oleuropein aglycone, dialdehyde form (OAD); 4: Decarboxymethyl ligstroside aglycone, oxidized dialdehyde form (oxidized phenolic compound 2) (DLAOD); 5: Ligstroside aglycone, dialdehyde form (LAD); 6: Decarboxymethyl ligstroside aglycone, dialdehyde form (DLAD); 7: Acetoxypinoresinol; 8: Oleuropein aglycone, oxidized aldehyde and hydroxylic form (oxidized phenolic compound 3) (OAOAH); and 9: Ligstroside aglycone, oxidized aldehyde and hydroxylic form (oxidized phenolic compound 4) (LAOAH)).

where: IA: The intensity of absorbance, mAU: milli-Absorbance Units, tR: Retention time.

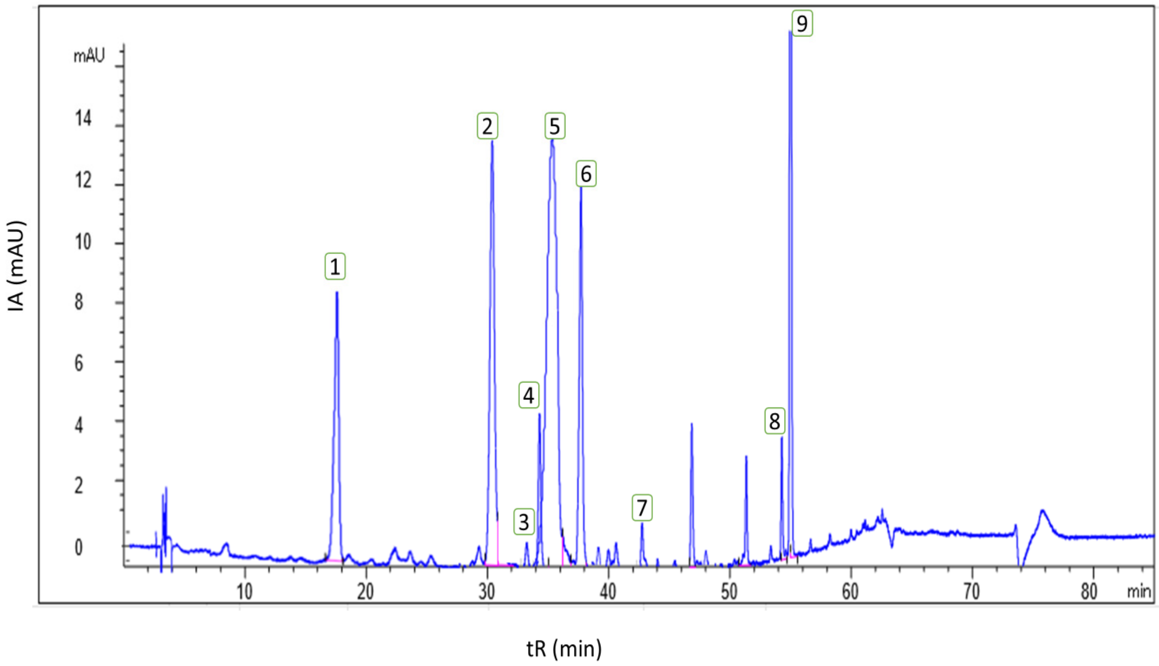

Figure S28. Total ion chromatograms (TICs) from the HPLC analysis of the phenolic compounds recorded at 280 nm in Experiment 24 (Manzanilla oil deep-fried at 210 °C for 48 h without polyphenols supplementation). 1: Internal standard (syringic acid); 2: Decarboxymethyl oleuropein aglycone, dialdehyde form (DOAD); 3: Oleuropein aglycone, dialdehyde form (OAD); 4: Ligstroside aglycone, dialdehyde form (LAD); 5: Decarboxymethyl ligstroside aglycone, oxidized dialdehyde form (oxidized phenolic compound 2) (DLAOD); 6: Decarboxymethyl ligstroside aglycone, dialdehyde form (DLAD); 7: Acetoxypinoresinol; 8: Oleuropein aglycone, oxidized aldehyde and hydroxylic form (oxidized phenolic compound 3) (OAOAH); and 9: Ligstroside aglycone, oxidized aldehyde and hydroxylic form (oxidized phenolic compound 4) (LAOAH)).

where: IA: The intensity of absorbance, mAU: milli-Absorbance Units, tR: Retention time.
